# Privacy-enhancing sequential learning under heterogeneous selection bias in multi-site electronic health records data

**DOI:** 10.1093/jamia/ocag083

**Published:** 2026-06-16

**Authors:** Ritoban Kundu, Maxwell Salvatore, Kumar Kshitij Patel, Lucila Ohno-Machado, Hyunghoon Cho, Xu Shi, Bhramar Mukherjee

**Affiliations:** Department of Biostatistics, Epidemiology & Informatics, University of Pennsylvania, Philadelphia, PA 19104, United States; Department of Biostatistics, Epidemiology & Informatics, University of Pennsylvania, Philadelphia, PA 19104, United States; Department of Genetics, University of Pennsylvania, Philadelphia, PA 19104, United States; Foundations of Data Science, Yale University, New Haven, CT 06511, United States; Biomedical Informatics & Data Science, Yale University, New Haven, CT 06510, United States; School of Medicine, Yale University, New Haven, CT 06510, United States; Biomedical Informatics & Data Science, Yale University, New Haven, CT 06510, United States; Department of Biostatistics, University of Michigan, Ann Arbor, MI 48109, United States; Department of Biostatistics, Yale University, New Haven, CT 06510, United States; Department of Chronic Disease Epidemiology, Yale University, New Haven, CT 06510, United States; Department of Statistics, Yale University, New Haven, CT 06511, United States

**Keywords:** decentralized learning, multiple robustness, electronic health records, sequential learning, selection bias

## Abstract

**Objectives:**

To develop privacy-enhancing statistical methods for estimating disease risk parameters across multiple electronic health record (EHR) sites with heterogeneous selection mechanisms, avoiding individual-level data sharing. We illustrate their utility via a cross-biobank analysis of smoking and 97 cancer subtypes using NIH All of Us (AOU) and Michigan Genomics Initiative (MGI) data sites.

**Materials and Methods:**

Distributed health platforms often render centralized algorithms infeasible due to patient privacy protection. We propose Sequential Pseudo-Likelihood (SPL) and Sequential Augmented Inverse Probability Weighting (SAIPW) to adjust for selection bias using summary statistics shared across sites and external population information. SAIPW employs flexible auxiliary models for multiple robustness. We compared SPL and SAIPW against unweighted and centralized/meta-learning benchmarks in simulations, applying them to harmonized MGI (*n* = 50 935) and AOU (*n* = 241 563) data.

**Results:**

Unweighted estimators exhibited substantial bias. SPL and SAIPW yielded unbiased estimates with valid coverage, with SAIPW remaining robust to selection model misspecification. Both approaches showed negligible efficiency loss relative to centralized methods. Meta-learning methods proved unstable for rare outcomes. Real-data analyses consistently identified strong associations between smoking and lung, bladder, and larynx cancers.

**Discussion:**

These findings highlight the necessity of adjusting for site-specific selection biases in distributed health networks. SPL and SAIPW offer practical, scalable solutions that bypass the instability of meta-analysis for rare events, successfully harmonizing diverse biobanks while strictly enhancing patient privacy.

**Conclusion:**

Our framework enables valid, privacy-enhancing inference across EHR sites subject to heterogeneous selection, facilitating scalable, distributed research using real-world data.

## Background and significance

**Selection bias and privacy constraints:** Electronic health records (EHRs) are foundational to clinical care and biomedical research, with adoption exceeding 96% of U.S. hospitals following the 2009 Health Information Technology for Economic and Clinical Health (HITECH) Act.^1–3^ Multi-site EHR analyses offer broader generalizability and greater statistical power. In particular, studying relatively rare conditions such as complications from invasive procedures, adverse drug events, or associations with rare genetic variants requires integrating data from multiple clinical sites to obtain accurate, generalizable, and reproducible results.

Integrating large biobanks is hindered by 2 key challenges: heterogeneous recruitment and privacy barriers that preclude centralized analysis. Recruitment strategies vary widely, from the disease-enriched surgical cohorts of the Michigan Genomics Initiative (MGI),^4^ to the demographically representative national cohort of the NIH All of Us Research Program (AOU).^5^ These differing mechanisms introduce cohort-specific selection bias,^6^ which can lead to biased association estimates if unaddressed.^7^^,^^8^ Simultaneously, data governance policies force initiatives like AOU, MGI, or the Million Veteran Program (MVP) to function as secure enclaves, making it difficult to pool raw individual-level data. These widespread challenges, evident in global efforts like the Global Biobank Meta-analysis Initiative (GBMI),^9^ underscore the urgent need for scalable, privacy-enhancing methods that enable valid distributed inference across heterogeneous EHR cohorts.

**Meta-analysis:** Among the available approaches for estimation under data-sharing constraints, meta-analysis remains a widely used and practical solution. The classical fixed-effects meta-analysis combines site-specific association estimates using inverse-variance weighting.^10^ While effective in many settings, this approach can perform poorly in unweighted generalized linear models^11^ or Inverse Probability Weighting (IPW) based causal estimators,^12^ particularly when individual sites have small sample sizes or rare outcome prevalence, leading to unstable or biased estimates.

**Federated and sequential methods:** Federated learning enables privacy enhancing multi-site analysis through the exchange of summary level statistics rather than individual level data. Likelihood based approaches^13–16^ provide scalable global parameter estimation under data sharing constraints. Similarly, estimating equation based distributed methods, such as renewable estimation^12^^,^^17^^,^^18^ are particularly effective for rare outcomes or small sample sizes. However, these methods typically assume site data are representative of the target population, ignoring site-specific sampling mechanisms. As a result, they may produce biased or non-generalizable estimates in the presence of selection bias, which is a frequent complication in multi-site EHR research.

**Our contribution:** To simultaneously address the challenges of privacy constraints and selection bias in multi-site EHR studies, we propose a sequential estimation framework. Building on the IPW and Augmented IPW (AIPW) estimating equations developed by Kundu et al,^19^ we embed these estimators within a renewable estimation paradigm suitable for distributed environments. Our methodology explicitly accommodates site-specific selection models and nuisance parameters while providing valid variance estimation. By leveraging external probability data representative of the target population, the proposed approach delivers robust, scalable, and privacy-enhancing inference. We evaluate its performance through comprehensive simulation studies and demonstrate its practical utility in a cancer Phenome-Wide Association Study (PheWAS) using data from the AOU and the MGI.

## Setup

### Problem setup

We aim to estimate the association between a binary disease indicator *D* and covariates $Z=(1,Z_{1},Z_{2},\ldots ,Z_{p})$ in a target population of size *N*. Specifically, we are interested in the estimation of $\theta_{Z}$ in the following disease risk model:


(1) $$\text{logit}(P(D=1|Z))=\theta_{Z}^{\prime }Z\cdot$$

**Figure 1.**
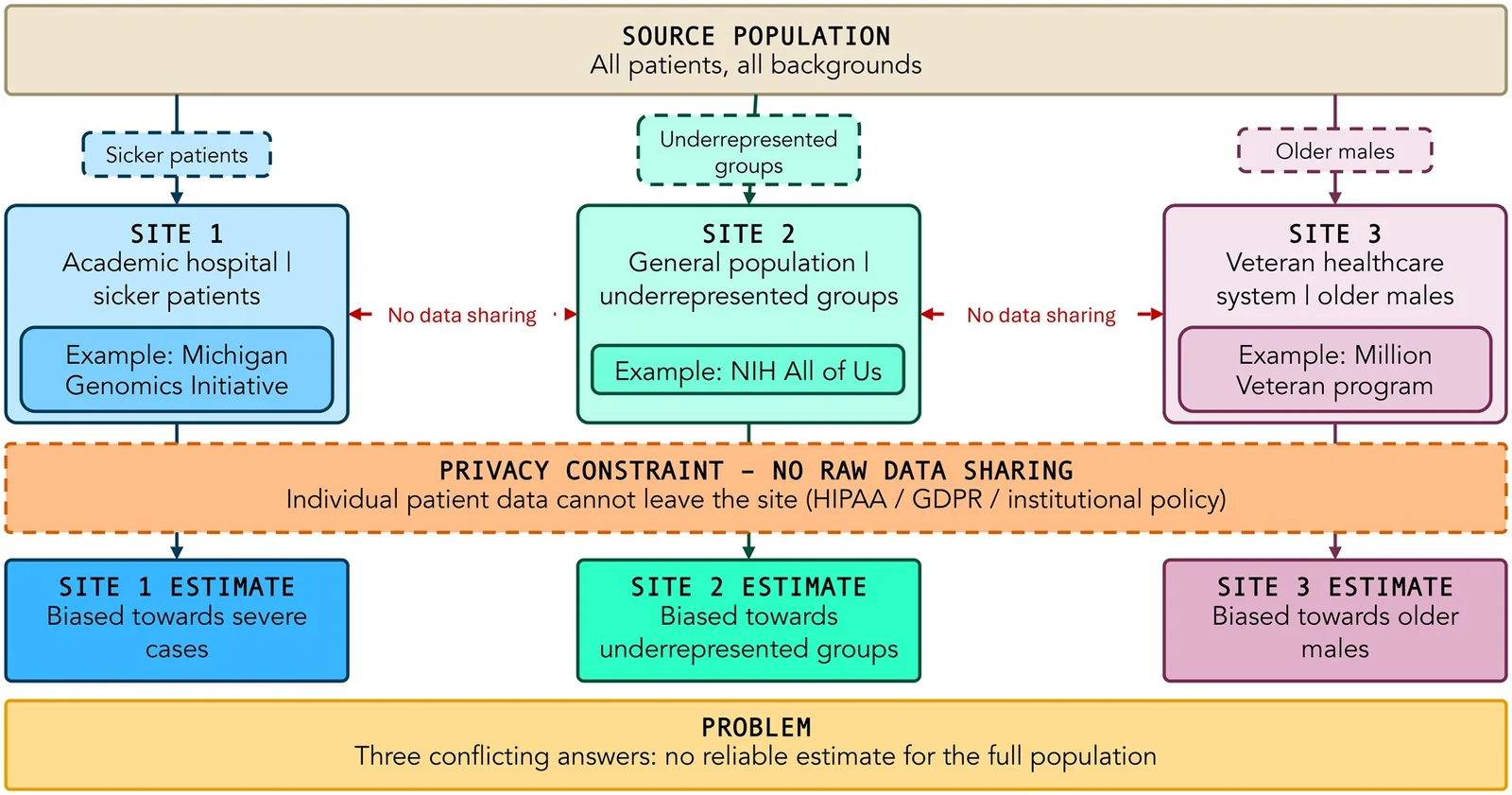
Motivating example with 3 EHR sites: the MGI, NIH AOU, and the MVP where each subject to site-specific selection bias toward distinct subpopulations (severely ill, under-represented groups, and older male patients, respectively). Privacy regulations (HIPAA/GDPR) prohibit sharing of individual-level data across sites, making standard pooled estimation infeasible.

Our target population constitutes a finite random sample drawn from a hypothetical super population. To estimate $\theta_{Z}$, we utilize data from *K* sites drawn from this target population, where each site k∈{1,2,…,K} is characterized by a binary selection indicator $S_{k}$. In practice, real world data from multiple EHR sites consistently exhibit highly heterogeneous selection mechanisms. Figure 1 motivates this challenge using 3 diverse sites: a hospital based biobank (MGI, $S_{1}=1$), a nationwide initiative sampling for underrepresented groups (AOU, $S_{2}=1$), and a veteran registry (MVP, $S_{3}=1$).

**Figure 2.**
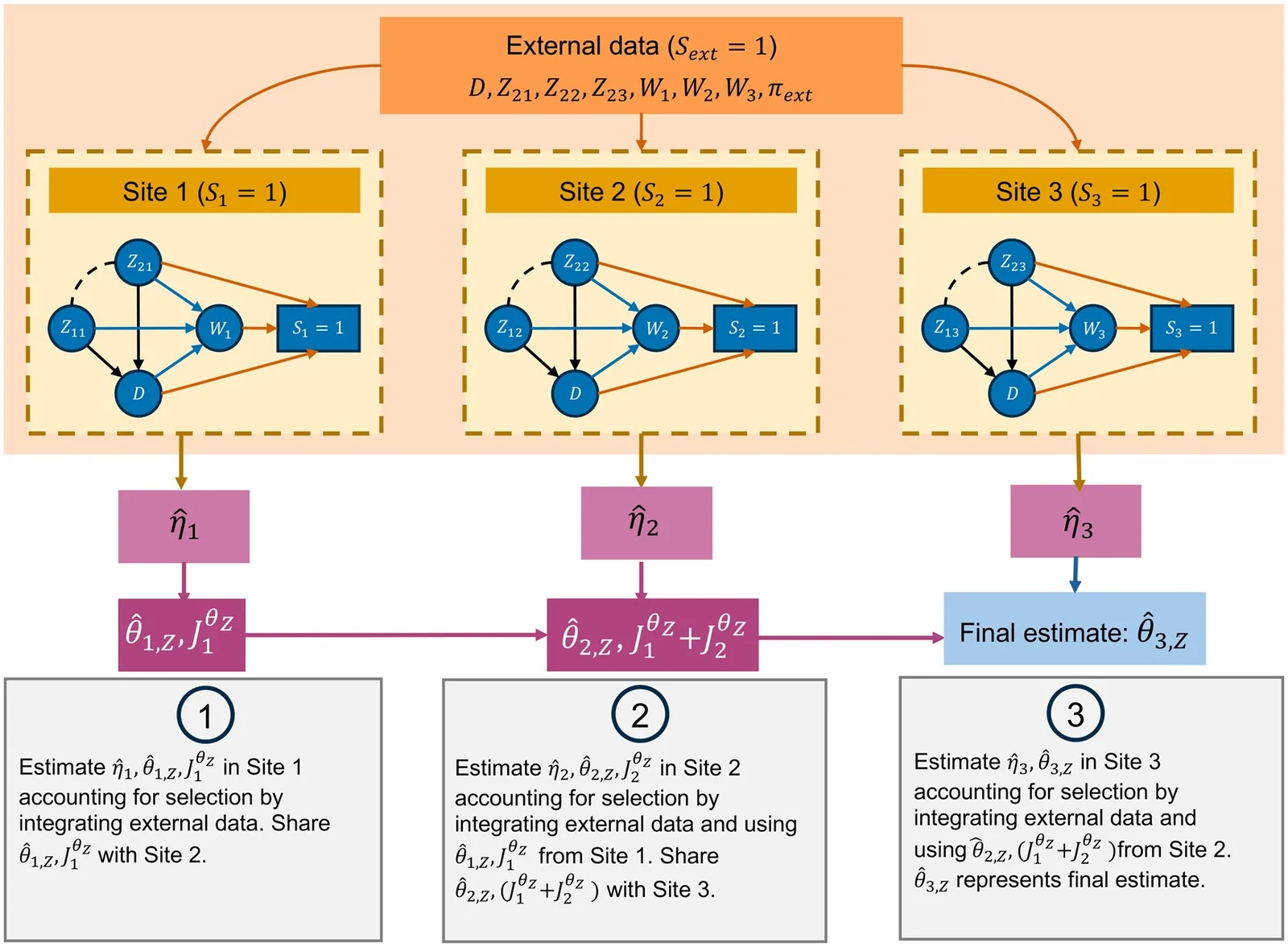
Illustration of the sequential algorithm with K=3 data sites for both the SPL and SAIPW methods. Each site sequentially estimates its local nuisance model parameters ${\hat{\eta }}_{k}$, and contributes to the global association parameter update ${\hat{\theta }}_{Z}$ by sharing only summary-level statistics, namely sensitivity matrix denoted by $J_{k}^{\theta_{Z}}$.

To formalize this heterogeneity, we partition Z into traits affecting only the disease ($Z_{1k}$) and those jointly affecting disease and selection ($Z_{2k}$), alongside unique site-specific selection drivers ($W_{k}$). Assuming disease *D* influences selection, the site specific probability is $P(S_{k}=1|X_{k})=\pi_{k}(X_{k})$, where $X_{k}=(D,Z_{2k},W_{k})$. Standard positivity ensures every individual in the target population has a positive probability of being selected at each site. Table 1 concretely maps these partitions for cancer (*D*) using age, race, income, and comorbidity as Z, while Figure 2 illustrates the relations between these variables in a setting with K=3 sites.

**Table 1.** Summary of variable definitions across the 3 EHR sites: the MGI, NIH All of Us (AOU), and the MVP.

Variable	General definition	Site 1: MGI	Site 2: AOU	Site 3: MVP
***D***	Target disease	Cancer	Cancer	Cancer
$Z_{1k}$	Traits affecting *only* the disease	Income	Comorbidity	Race, Comorbidity, Income
$Z_{2k}$	Traits affecting *both* disease and selection	Age, Race, Comorbidity	Age, Race, Income	Age
$W_{k}$	Traits affecting *only* selection	BMI	Sexual orientation	Military deployment history

Variables are categorized: disease-only covariates ($Z_{1k}$), shared predictors of both disease and site covariates ($Z_{2k}$), and selection-only covariates ($W_{k}$).

Moreover, strict institutional privacy constraints prohibit sharing individual level raw data across sites, rendering centralized statistical pooling impossible. Consequently, we develop a suite of privacy enhancing sequential estimators designed to correct for these diverse selection biases utilizing strictly aggregate summary statistics.

## Methods

In this section, we present methods for estimation of $\theta_{Z}$ under multi-site heterogeneous selection bias and privacy barriers. Beyond the baseline unweighted logistic regression, we propose the IPW based Sequential Pseudo-Likelihood (SPL) method and the multiply robust Sequential AIPW (SAIPW) approach. We first define the theoretical centralized oracle for each method to benchmark efficiency loss in our simulations. While demonstrated via logistic regression, our methodology extends to any disease model characterized by a valid parametric estimating equation.

### Unweighted logistic regression

#### Centralized unweighted logistic method (CUW)

This method assumes individual-level data can be stacked from all sites. For a standard logistic regression of outcome *D* on Z, the individual score contribution for subject *i* using Maximum Likelihood Estimation (MLE) is:


(2) $$T_{i}(\theta_{Z})=D_{i}Z_{i}-\frac{e^{\theta_{Z}^{\prime }Z_{i}}}{1+e^{\theta_{Z}^{\prime }Z_{i}}}Z_{i}\cdot$$

Next, the score function for a specific site *k* is constructed by aggregating the individual contributions of its members:


(3) $$U_{k}(\theta_{Z})=\frac{1}{N}\sum_{i=1}^{N}S_{ki}T_{i}(\theta_{Z})\cdot$$

The final CUW estimating equation is obtained by combining the score functions across all *K* sites:


(4) $$\sum_{k=1}^{K}U_{k}(\theta_{Z})=0\cdot$$

This estimator for $\theta_{Z}$ can be **substantially biased** when site-specific selection mechanisms are present.^19^

#### Sequential unweighted logistic regression (SUW)

Directly adopting the renewable estimation framework of Luo et al,^17^ SUW sequentially aggregates summary statistics across sites to estimate parameters and variance. Without site-specific selection adjustments, it serves as our baseline sequential estimator.

### Weighted logistic regression

#### Centralized pseudo-likelihood (CPL)

This method assumes a hypothetical scenario where individual-level data from all sites are stacked together. To adjust for selection bias we adapt a IPW based Pseudo-Likelihood (PL) approach.^20^ Let $\alpha_{k}$ parameterize the selection model for site *k*, namely $\pi_{k}(X_{ki})=\pi_{k}(X_{ki},\alpha_{k})$. The IPW-adjusted score function for the *k*th site is:


(5) $$U_{k}(\theta_{Z},\alpha_{k})=\frac{1}{N}\sum_{i=1}^{N}\frac{S_{ki}}{\pi_{k}(X_{ki},\alpha_{k})}T_{i}(\theta_{Z}),$$

where $T_{i}(\theta_{Z})$ is defined by Equation (2). Selection probabilities $\pi_{k}(\cdot )$ are estimated by integrating an external individual-level probability sample, where $S_{\text{ext}}$ denotes the indicator variable for selection into the external data. An example of an external individual-level probability sample is the National Health Interview Survey (NHIS). For each data site k∈{1,2,…,K}, ${\hat{\alpha }}_{k}$ are obtained via the PL method, by solving:


(6) $$\frac{1}{N}\sum_{i=1}^{N}S_{ki}X_{ki}-\frac{1}{N}\sum_{i=1}^{N}\left(\frac{S_{\text{ext},i}}{\pi_{\text{ext},i}}\right)\pi_{k}(X_{ki},\alpha_{k})X_{ki}=0,$$

where $\pi_{\text{ext}}$ represents the known inclusion probabilities from the external sample. The final CPL estimating equation, aggregating across all sites, is given by:


(7) $$\sum_{k=1}^{K}U_{k}(\theta_{Z},{\hat{\alpha }}_{k})=0,$$

This method yields unbiased estimates provided the selection models for all sites are correctly specified.^19^

#### Proposal 1: sequential pseudo-likelihood (SPL)

This privacy-enhancing estimator extends the CPL framework to distributed settings by eliminating the need for individual-level data sharing. The process begins at the first site, which estimates its local selection parameter ${\hat{\alpha }}_{1}$ and the initial association parameter ${\hat{\theta }}_{1,Z}$. It then transmits ${\hat{\theta }}_{1,Z}$ to the second site, alongside its local sensitivity matrix $J_{1}^{\theta_{Z}}({\hat{\theta }}_{1,Z},{\hat{\alpha }}_{1})$, defined as the site-specific negative gradient of the IPW estimating function from Equation (5) with respect to the target parameter $\theta_{Z}$. Intuitively, this matrix reflects how informative the local data are about the association parameter. Each subsequent site k>1 estimates its own local selection parameter ${\hat{\alpha }}_{k}$ and updates the overall association parameter ${\hat{\theta }}_{k,Z}$ via a renewable estimating equation. This update utilizes the prior estimate ${\hat{\theta }}_{k-1,Z}$ and the cumulative sensitivity matrix $\sum_{m=1}^{k-1}J_{m}^{\theta_{Z}}({\hat{\theta }}_{m,Z},{\hat{\alpha }}_{m})$, which serves as a first-order proxy for all historical estimating equations. This allows each new site to incorporate prior information without accessing individual-level data from previous sites. The formal mathematical procedure is detailed in Algorithm 1, and the sequential workflow is illustrated in Figure 2.

Algorithm 1: Sequential Pseudo-Likelihood Algorithm

**Definitions:** 

Site-specific sensitivity matrix of IPW estimating function from Equation (5),

$$J_{k}^{\theta_{Z}}(\theta_{Z},\alpha_{k})=-{\frac{\partial U_{k}(\theta_{Z},\alpha_{k})}{\partial \theta_{Z}}|}_{\theta_{Z}=\theta_{Z}}$$

**Initialization (Site 1):** 

1. Estimate local selection parameter ${\hat{\alpha }}_{1}$.
2. Compute initial association parameter estimate ${\hat{\theta }}_{1,Z}$ by solving local estimating equation $U_{1}(\theta_{Z},{\hat{\alpha }}_{1})=0$.
3. Calculate local sensitivity matrix $J_{1}^{\theta_{Z}}({\hat{\theta }}_{1,Z},{\hat{\alpha }}_{1})$.
4. Transmit $\left\{{\hat{\theta }}_{1,Z},J_{1}^{\theta_{Z}}({\hat{\theta }}_{1,Z},{\hat{\alpha }}_{1})\right\}$ to Site 2.

**Sequential Updating (Sites**  k=2  **to *K*):** 

1. Receive prior association parameter estimate ${\hat{\theta }}_{k-1,Z}$ and cumulative sensitivity matrices $\sum_{m=1}^{k-1}J_{m}^{\theta_{Z}}({\hat{\theta }}_{m,Z},{\hat{\alpha }}_{m})$ from Site k−1.
2. Estimate local selection parameter ${\hat{\alpha }}_{k}$.
3. Obtain updated association parameter estimator ${\hat{\theta }}_{k,Z}$ by solving the renewable estimating equation:
  $$\sum_{m=1}^{k-1}J_{m}^{\theta_{Z}}({\hat{\theta }}_{m,Z},{\hat{\alpha }}_{m})\left({\hat{\theta }}_{k-1,Z}-\theta_{Z}\right)+U_{k}(\theta_{Z},{\hat{\alpha }}_{k})=0$$
4. Calculate local sensitivity matrix $J_{k}^{\theta_{Z}}({\hat{\theta }}_{k,Z},{\hat{\alpha }}_{k})$ and update cumulative sum.
5. If k<K, transmit updated $\left\{{\hat{\theta }}_{k,Z},\sum_{m=1}^{k}J_{m}^{\theta_{Z}}({\hat{\theta }}_{m,Z},{\hat{\alpha }}_{m})\right\}$ to Site k+1.

**Final Output:** 

Site *K* produces the final, association parameter estimate ${\hat{\theta }}_{K,Z}$.

**Privacy implications for estimation:** Sequentially transmitting updated association parameters and cumulative weighted sensitivity matrices avoids sharing raw data. Crucially, because selection weights are constructed using already public external data, this approach introduces no additional disclosure risk relative to SUW. However, sharing aggregate statistics carries theoretical risks even for SUW. In edge cases with extremely small sample sizes or sparse covariates, these aggregates could potentially be targeted for membership inference, group level property inference, or gradient based reconstruction attacks, though the direct leakage of individual level records remains highly unlikely.

**Note:** Variance estimation procedures and privacy considerations for CPL and SPL are detailed in Sections S2.1 and S2.2. To address the susceptibility to bias that arises when a site specific selection model is misspecified, we propose a multiply robust framework.

### Multiply robust method

#### Centralized augmented inverse probability weighting (CAIPW)

To protect against selection model misspecification, we modify the AIPW method of Kundu et al,^19^ to specifically address scenarios where $W_{j}$ is unavailable in site *i* (i≠j). The CAIPW score augments the standard IPW estimator by incorporating an auxiliary score model using external data, yielding a multiply robust estimating equation. The auxiliary score model is defined by the projection of the unweighted logistic score, $T_{i}(\theta_{Z})$ onto $X_{k}$,


$$f_{k}(X_{ki},\theta_{Z})=E[T_{i}(\theta_{Z})|X_{ki}]$$

Even if the selection model is completely misspecified, the CAIPW estimator remains unbiased provided this auxiliary score model is correct. In sites where selection is independent of *D* conditional on $X_{k}=(Z_{2k},W_{k})$, we can define the auxiliary score model using the conditional model, namely $D|X_{k}$.

Let ${\hat{\eta }}_{k}=({\hat{\alpha }}_{k},{\hat{f}}_{k})$ denote the estimated combined nuisance parameters for site *k*. We estimate ${\hat{\alpha }}_{k}$ using PL and ${\hat{f}}_{k}$ using XGBoost. To prevent overfitting from these complex machine learning algorithms, we employ a rigorous double machine learning (DML) based cross fitting strategy, which is detailed fully in the Section S1.

Utilizing ${\hat{\eta }}_{k}$, the CAIPW method constructs the site-specific augmented score function:


(8) $$U_{k}(\theta_{Z},{\hat{\eta }}_{k})=\frac{1}{N}\sum_{i=1}^{N}\frac{S_{ki}}{\pi_{k}(X_{ki},{\hat{\alpha }}_{k})}\left(T_{i}(\theta_{Z})-{\hat{f}}_{k}(X_{ki},\theta_{Z})\right)+\frac{1}{N}\sum_{i=1}^{N}\frac{S_{\text{ext},i}}{\pi_{\text{ext},i}}{\hat{f}}_{k}(X_{ki},\theta_{Z})\cdot$$

The final CAIPW framework simply aggregates these site-specific components across all sites to formulate the multiply robust estimating equation:


(9) $$\sum_{k=1}^{K}U_{k}(\theta_{Z},{\hat{\eta }}_{k})=0\cdot$$

#### Proposal 2: sequential augmented inverse probability weighting (SAIPW)

To provide multiply-robust protection without pooling individual-level data, the SAIPW estimator translates the centralized CAIPW framework into a sequential process. The computational workflow establishes its baseline at the first site through the estimation of both the joint local nuisance parameters ${\hat{\eta }}_{1}$ and the association parameter ${\hat{\theta }}_{1,Z}$. This site communicates ${\hat{\theta }}_{1,Z}$ forward, paired with a local sensitivity matrix $J_{1}^{\theta_{Z}}({\hat{\theta }}_{1,Z},{\hat{\eta }}_{1})$, derived as the negative gradient of the AIPW estimating function from Equation (8). For every following site k>1, the association parameter estimate is updated to ${\hat{\theta }}_{k,Z}$ via a renewable estimating equation, following the calculation of local nuisance parameters ${\hat{\eta }}_{k}$. This renewable update strategically merges the prior estimate ${\hat{\theta }}_{k-1,Z}$ with the cumulative sensitivity matrix $\sum_{m=1}^{k-1}J_{m}^{\theta_{Z}}({\hat{\theta }}_{m,Z},{\hat{\eta }}_{m})$. Algorithm 2 details this mathematical procedure, and Figure 2 provides a visual summary.

Algorithm 2: Sequential Augmented Inverse Probability Weighting (SAIPW)

**Definitions:** 

Site-specific sensitivity matrix of AIPW estimating function from Equation (8),

$$J_{k}^{\theta_{Z}}(\theta_{Z},\eta_{k})=-{\frac{\partial U_{k}(\theta_{Z},\eta_{k})}{\partial \theta_{Z}}|}_{\theta_{Z}=\theta_{Z}}$$

**Initialization (Site 1):** 

1. Estimate local nuisance parameters ${\hat{\eta }}_{1}$.
2. Compute ${\hat{\theta }}_{1,Z}$ by solving $U_{1}(\theta_{Z},{\hat{\eta }}_{1})=0$.
3. Calculate $J_{1}^{\theta_{Z}}({\hat{\theta }}_{1,Z},{\hat{\eta }}_{1})$.
4. Transmit $\left\{{\hat{\theta }}_{1,Z},J_{1}^{\theta_{Z}}\right\}$ to Site 2.

**Sequential Updating (Sites**  k=2,…,K**):** 

1. Receive ${\hat{\theta }}_{k-1,Z}$ and $\sum_{m=1}^{k-1}J_{m}^{\theta_{Z}}({\hat{\theta }}_{m,Z},{\hat{\eta }}_{m})$ from Site k−1.
2. Estimate local nuisance parameters ${\hat{\eta }}_{k}$.
3. Obtain ${\hat{\theta }}_{k,Z}$ by solving the renewable augmented estimating equation:
  $$\sum_{m=1}^{k-1}J_{m}^{\theta_{Z}}({\hat{\theta }}_{m,Z},{\hat{\eta }}_{m})\left({\hat{\theta }}_{k-1,Z}-\theta_{Z}\right)+U_{k}(\theta_{Z},{\hat{\eta }}_{k})=0$$
4. Calculate $J_{k}^{\theta_{Z}}({\hat{\theta }}_{k,Z},{\hat{\eta }}_{k})$.
5. If k<K, transmit $\left\{{\hat{\theta }}_{k,Z},\sum_{m=1}^{k}J_{m}^{\theta_{Z}}\right\}$ to Site k+1.

**Final Output:** 

Site *K* outputs the fully updated, multiply-robust estimator ${\hat{\theta }}_{K,Z}$.

**Privacy implications for estimation:** While the SAIPW method transmits the same aggregate quantities as SPL, the complex functional forms of its machine learning auxiliary models offer comparatively greater privacy protection. This algorithmic complexity renders the shared matrices analytically more opaque, providing enhanced safeguards against gradient based vulnerabilities for the underlying site data.

**Note:** Detailed variance estimation and privacy implications for CAIPW and SAIPW are provided in Sections S2.3 and S2.4. These estimators are multiply robust, meaning consistent estimation of disease model parameters is guaranteed if either the selection model $\pi_{k}(\cdot )$ or the auxiliary model $f_{k}(\cdot )$ is correctly specified for every site *k*.

**Communication cost for point estimation:** Regarding communication efficiency during the point estimation phase, both SPL and SAIPW require transmitting strictly a (p+1) dimensional parameter vector and a (p+1)×(p+1) cumulative sensitivity matrix, where *p* is the dimension of the covariates. Consequently, the communication payload from one site to the next is $O({\left(p+1\right)}^{2})+O(p+1)$, resulting in an overall transmission cost of $O(p^{2})$. Detailed communication costs for the variance estimation of SPL and SAIPW are provided in Section S2.4. Table 2 provides a practical comparison of these sequential methods and their code availability. Additionally, a comprehensive list of abbreviations is provided in Table S1 to facilitate ease of reference.

**Table 2.** Comparison of 3 sequential estimation methods: SUW, SPL, and SAIPW, summarizing their methodological features, code availability, and relative strengths and weaknesses.

	SUW sequential unweighted	SPL sequential pseudo-likelihood	SAIPW sequential augmented IPW
**Description**	Unweighted logistic regression Sequentially implemented across sites Shares only cumulative sensitivity matrix and association parameter estimates for both estimation and variance estimation.	IPW regression via Pseudo Likelihood Integrates external probability samples for selection weights Sequentially implemented across sites Shares parameters and cumulative sensitivity matrix for estimation. Shares additional supplementary matrices for variance estimation.	IPW + auxiliary score model (XGBoost) Uses external data for nuisance parameter models Sequentially implemented across sites Pseudo Likelihood + XgBoost + cross fitting (DML) Shares parameters and cumulative sensitivity matrix for estimation. Shares additional supplementary matrices for variance estimation.
**Code**	github.com/luolsph/RenewGLM_pkg/	github.com/Ritoban1/Collaborative-Learning/	github.com/Ritoban1/Collaborative-Learning
**Pros**	Fastest implementation time Enhanced privacy vs centralized counterpart Shares least number of summary statistics for variance estimation	Accounts for site-specific selection bias Enhanced privacy vs centralized counterpart Faster than SAIPW implementation Better than meta PL for small samples	Accounts for site-specific selection bias Multiply robust: protect against selection model misspecification Enhanced privacy vs centralized counterpart Complex nuisance parameter models enhances privacy compared to SPL
**Cons**	Substantially biased with selection bias Bias amplified in multi-site sampling	Heavily biased if selection model misspecified High variance near positivity violations Least privacy preserving in case of variance estimation Requires individual-level external data	Computationally intensive (ML-based) Less stable for small or rare samples than SPL due to ML auxiliary score models Requires individual-level external data

## Simulation

### Setup

We simulate a target population of N=100,000 divided into 3 disjoint subpopulations, from which 3 EHR sites are sampled (n≈ 14 000, 12 000, and 11 000). An external probability sample of 30 000 is also generated. Covariates $Z_{1},Z_{2},Z_{3}$ follow a tri-variate Gaussian distribution. *D* is generated via:


(10) $$\text{logit}(P(D=1|Z_{1},Z_{2},Z_{3}))=-2+0.35Z_{1}+0.45Z_{2}+0.35Z_{3}\cdot$$

Conditional on $D,Z_{1},Z_{2}$, and $Z_{3}$, the variables $W_{1},W_{2}$, and $W_{3}$ are drawn from site-specific Gaussian distributions. The selection variables for each site are defined as $X_{1}=(D,W_{1},Z_{2},Z_{3})$, $X_{2}=(D,W_{2},Z_{3})$, and $X_{3}=(D,W_{3},Z_{2})$.

### Model fitting and metrics of comparison

We compared 3 approaches for the UW, PL, and AIPW estimators: (i) Centralized (pooled oracle benchmark); (ii) Sequential (proposed); and (iii) Meta-Analysis (fixed-effects via the meta R package^21^). For AIPW, XGBoost was utilized to estimate auxiliary scores. We assessed robustness across 3 selection model scenarios as detailed in Table 3: (i) correctly specified; (ii) misspecified Scenario 1 (omission of *D*); and (iii) misspecified Scenario 2 (incorporating heterogeneous omissions of selection variables). Performance was evaluated over R=500 replications using the following metrics:

**Table 3.** Site-specific selection models used in simulations, including the true data-generating model, the correctly specified fitted model, and 2 misspecified fitted models omitting the disease indicator and heterogeneous selection variables, respectively.

Site	Actual selection variables	Correctly specified	Misspecified Scenario 1	Misspecified Scenario 2
1	$D,W_{1},Z_{2},Z_{3}$	$D,W_{1},Z_{2},Z_{3}$	$W_{1},Z_{2},Z_{3}$	$W_{1},Z_{2}$
2	$D,W_{2},Z_{3}$	$D,W_{2},Z_{3}$	$W_{2},Z_{3}$	$W_{2}$
3	$D,W_{3},Z_{2}$	$D,W_{3},Z_{2}$	$W_{3},Z_{2}$	$D,Z_{3}$

1. Relative Bias Percentage (RBP):
  $$\text{RBP}=\left|\frac{1}{R}\sum_{r=1}^{R}\frac{{\hat{\theta }}_{r}-\theta }{\theta }\right|\times 100.$$
2. Relative Mean Squared Error (RMSE):
  $$\text{RMSE}=\frac{\sum_{r=1}^{R}{\left({\hat{\theta }}_{r}-\theta \right)}^{2}}{\sum_{r=1}^{R}{\left({\hat{\theta }}_{\text{naive},r}-\theta \right)}^{2}}.$$
3. Relative Efficiency (RE):
  $$\text{RE}=\frac{\sum_{r=1}^{R}{\left({\hat{\theta }}_{\text{seq}/\text{meta},r}-\theta \right)}^{2}}{\sum_{r=1}^{R}{\left({\hat{\theta }}_{\text{cent},r}-\theta \right)}^{2}}.$$
4. Coverage probabilities.

### Results

**Figure 3.**
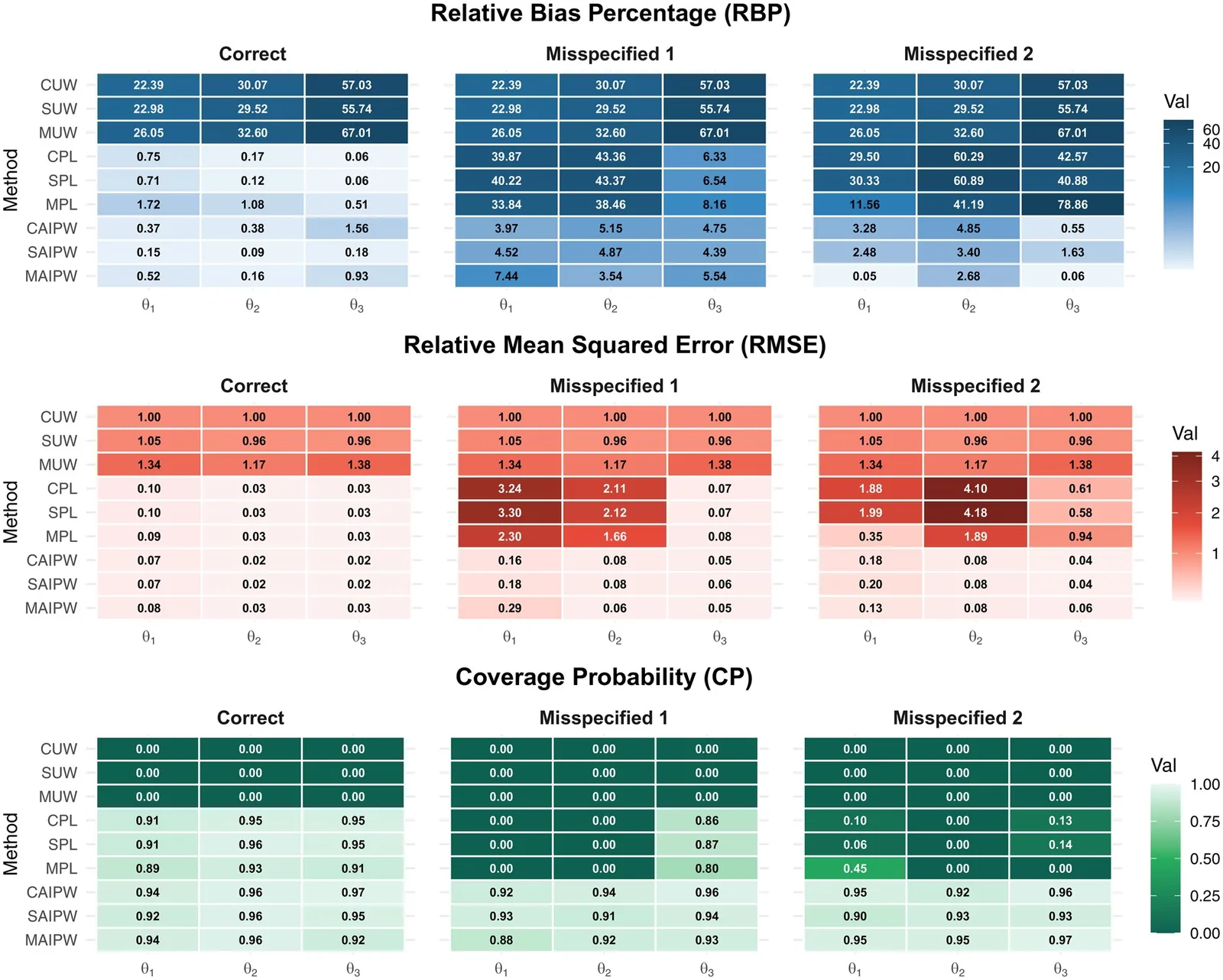
Performance metrics of unweighted, centralized, sequential, and meta-analysis estimators across 3 selection model specification scenarios. Results are presented for RBP, RMSE, and CP for target parameters $\theta_{1}$, $\theta_{2}$, and $\theta_{3}$. The scenarios include a correctly specified model, Misspecified Scenario 1 (omission of *D*), and Misspecified Scenario 2 (heterogeneous omission of selection variables).

The simulation results are presented in Figure 3.

#### Unweighted methods (CUW, SUW, MUW)

Unweighted methods, ignoring selection bias, exhibit high bias (RBP 22%-67%) and elevated RMSE across all scenarios. Consequently, coverage probabilities remain uniformly zero across all unweighted approaches regardless of model specification.

#### Centralized PL and AIPW estimators (CPL, CAIPW)

Centralized estimators serve as benchmarks. Under correct specification, CPL and CAIPW yield low bias and nominal coverage (≈ 0.95). Under both misspecification scenarios, CPL degrades significantly, whereas CAIPW remains robust, demonstrating the protective benefit of multiple robustness even when key variables are omitted in the selection models.

#### Sequential PL and AIPW estimators (SPL, SAIPW)

Under correct specification, both methods perform strongly with minimal bias and nominal coverage. Under misspecification, SPL degrades distinctly: bias reaches 43% in Scenario 1 and worsens to 60% for $\theta_{2}$ in the Misspecified Scenario 2, with coverage dropping near zero. In contrast, SAIPW remains highly robust to selection model misspecification. Even when outcomes and key auxiliary covariates are heterogeneously omitted (Scenario 2), SAIPW successfully mitigates bias (RBP <5%) and maintains valid coverage (0.90-0.93). This confirms its strong multiple robustness with negligible efficiency loss compared to centralized benchmarks.

#### Meta PL and AIPW estimators (MPL, MAIPW)

In large samples, they match centralized benchmarks under correct specification. However, under misspecification, their performance is mixed and occasionally unstable (eg, MPL bias reaching 79% for $\theta_{3}$ in Scenario 2, and MAIPW coverage dropping to 88% for $\theta_{1}$ in Scenario 1). This variability highlights the limitations of classical fixed-effect variance estimators when the site independence assumption is violated by the use of shared external data.

### Additional simulations: Large *K*

We evaluated CPL, SPL, and MPL, alongside unweighted benchmarks, in a setting characterized by many small sites (K=20; n∈[230,342]) with rare disease prevalence (3.4%-9.7%). We exclude the AIPW estimator because the cross-fitting required to train complex, site-specific machine learning models often results in data folds with zero or extremely few cases, leading to severe numerical instability. An external probability sample of size 2000 was used. The site-specific selection indicator $S_{k}$ depended on $D,Z_{2},Z_{3}$ and a site-specific $W_{k}$. In addition to a correctly specified model, we assessed performance under a heterogeneous misspecification scenario where *D* was omitted for the first 10 sites and $W_{k}$ were omitted for the remaining 10 sites during model fitting.

**Results:** In the setting with numerous small sites (K=20, Figure S1), unweighted methods exhibit severe bias, exceeding 77% RBP for $\theta_{3}$. Even under correct specification, MPL yields extremely high bias (89% RBP for $\theta_{3}$) and invalid coverage (0.31), degrading further to 100% bias under misspecification. Conversely, our proposed SPL matches the centralized CPL benchmark perfectly. Both achieve negligible bias and nominal coverage near 0.95 under correct specification. Under heterogeneous misspecification, both CPL and SPL predictably degrade, showing moderate bias increases and the elevated variance typical of small sample settings. Even with this expected degradation, SPL significantly outperforms MPL, maintaining strong alignment with the centralized benchmark.

### Simulation results summary

Unweighted methods exhibit high bias and RMSEs coverage across all scenarios by ignoring heterogeneous selection mechanisms. Our proposed sequential estimators achieve efficiency comparable to centralized benchmarks. Notably, while meta-analysis approaches perform well in large sample settings, they fail in scenarios with numerous small sites. In contrast, SPL matches the oracle perfectly even in these challenging small sample environments. Furthermore, while SPL degrades under misspecification, the multiply robust SAIPW framework provides protection against selection model misspecification, though it may become unstable in extremely small sample settings where data scarcity hinders fitting of site-specific complex machine learning models.

## Cancer PheWAS application: association between smoking and cancer types

### Introduction to data

**Setup and goal:** We conduct a PheWAS to investigate the relationship between smoking and a wide range of cancer types using 2 major EHR datasets: MGI and AOU. Smoking is a known risk factor for various cancers, including those of the blood (eg, acute myeloid leukemia), bladder, cervix, colon, esophagus, kidney, larynx, liver, lung, mouth, pancreas, pharynx, stomach, and trachea (CDC Smoking-Cancer). Our goal is to empirically evaluate these associations using rich EHR-linked sites while addressing the dual challenges of site specific selection bias and strict data governance policies that prevent raw individual level data sharing.

**Data sources:** The AOU cohort consists of 241 563 individuals from the CDR V8 release, reflecting a broad demographic reach through intentional oversampling of underrepresented groups.^5^ In contrast, the MGI cohort includes 50 935 individuals recruited mainly from preoperative anesthesia visits, providing a clinically enriched site.^4^ To adjust for selection mechanisms, we incorporate the 2019 National Health Interview Survey (NHIS) as a nationally representative external reference (*N* = 27 152).

### Data summary

**Cancer data summary:** We began with 100 cancer-related phecodes for our PheWAS. The full list of phecodes is provided in Section S3 for reference. We excluded 3 phecodes, namely “149.5,” “196,” and “197” due to a lack of cases in the AOU dataset. In the AOU cohort, the overall prevalence of any cancer diagnosis was 23.35%. Prevalence estimates for individual cancer types ranged from 0.02% for phecode “149.3” (indicating a rare malignancy of the pharynx) to 6.44% for phecode “195” (representing cancer, unspecified site). In contrast, the MGI cohort exhibited a substantially higher overall cancer prevalence of 52.36%, reflective of its clinically enriched recruitment design. In this cohort, prevalence ranged from 0.06% for phecode “204.3” (representing chronic lymphocytic leukemia) to 38.14% for phecode “195.”

**Summary of cohort characteristics:** Detailed in Table 4, the 3 cohorts show distinct profiles. MGI is characterized by an older, predominantly non-Hispanic White population with higher comorbidity burdens. AOU includes a higher proportion of females and sexual minorities than the nationally representative NHIS. Smoking rates were highest in the clinically enriched MGI cohort (49.28%) and lowest in NHIS (36.69%). These heterogeneous characteristics across the 2 EHR linked sites and the external probability sample underscore the need for the selection adjustment methods proposed in this study.

**Table 4.** Summary statistics of key demographic and clinical variables across the MGI, AOU, and NHIS cohorts.

Variables	MGI	AOU	NHIS (weighted)
**Sample size**	50 935	241 563	27 152
**Age**	Mean: 58.98 SD: 16.59.	Mean: 55.04 SD: 17.18.	Mean: 47.97 SD: 18.25.
**Biological sex**	Females: 53.07%	Female: 61.93%	Females: 50.89%
**Race**	NHW: 89.13%	NHW: 63.87%	NHW: 66.05%
**Overall cancer**	Yes: 52.36%	Yes: 23.35%	Yes: 9.64%
**Smokers**	Ever smoked: 49.28%	Ever smoked: 39.87%	Ever smoked: 36.69%
**Type 2 diabetes**	Yes: 30.85%	Yes: 22.72%	Yes: 8.93%
**CHD**	Yes: 17.18%	Yes: 14.26%	Yes: 4.61%
**BMI**	Mean: 29.86 SD: 7.06.	Mean: 30.08 SD: 7.77.	Mean: 27.85 SD: 5.48.
**Sexual orientation**	NA	Not straight: 11.17%	Not straight: 3.10%
**Health insurance**	NA	Yes: 96.01%	Yes: 88.87%
**Income**	NA	Over 75K: 39.14%	Over 75K: 45.00%
**Region**	NA	West: 24.79% South: 17.14% Northeast: 30.10% Midwest: 21.43%	West: 23.13% South: 37.58% Northeast: 17.85% Midwest: 21.43%

Reported values include sample sizes, means and standard deviations for continuous variables, and percentages for categorical variables. NHIS values are weighted using survey weights to reflect population-level estimates. Here, CHD stands for Coronary Heart Disease.

### Model fitting characteristics

#### Cancer PheWAS model

For each of the 97 cancer phecodes, we estimated the conditional association between smoking and cancer risk using logistic regression, targeting the U.S. adult population adjusted for biological sex, age and race. Let $D_{c}\in \{0,1\}$ denote the binary indicator for the presence of cancer type c∈{1,2,…,97}. For each cancer type, we aim to estimate the target parameter $\beta_{1,c}$ in the following model:


$$\begin{array}{c} \text{logit}\left\{P(D_{c}=1|\text{Smoke},\text{AgeCat},\text{Sex},\text{Race})\right\}=\beta_{0,c}+\beta_{1,c}I(\text{Smoke}=1)+\sum_{j=1}^{3}\beta_{2j,c}I(\text{AgeCat}=j)+\beta_{3,c}I(\text{Sex}=\text{Female})+\beta_{4,c}I(\text{Race}=\text{NHW}), \end{array}$$

where j=1,2,3 denotes age categories of the categorized age variable “AgeCat” corresponding to 35–49, 50–64, and 65+ years; (reference: 18–34 years).

#### Selection models

**AOU:** For the AOU cohort, the selection model incorporated age, biological sex, race, presence of health insurance (HI), sexual orientation (SO; non-straight vs straight), annual household income above $75 000 (IN), and geographic region (Northeast, South, Midwest, West).^5^ We assume the following selection model:


$$\begin{array}{c} \text{logit}\left\{P(S_{\text{AOU}}=1|\text{Smoke},\text{AgeCat},\text{Sex},\text{Race},\text{HI},\text{SO},\text{IN},\text{Region})\right\}\\ =\alpha_{10}+\alpha_{11}I(\text{Smoke}=1)+\sum_{j=1}^{3}\alpha_{1(1+j)}I(\text{AgeCat}=j)+\alpha_{15}I(\text{Sex}=\text{Female})+\alpha_{16}I(\text{Race}=\text{NHW})+\alpha_{17}\cdot I(\text{HI}=1)+\alpha_{18}I(\text{SO}=\text{Non-straight})+\alpha_{19}I(\text{IN}>75k)+\sum_{r=1}^{3}\alpha_{1(9+r)}I(\text{Region}=r), \end{array}$$

where Region=r denotes Northeast, Midwest, and South, with West as the reference category.

**MGI:** The selection model for the MGI cohort included age, coronary heart disease (CHD), type 2 diabetes (DI), race (Non-Hispanic White, NHW), history of cancer (C), and smoking.^8^^,^^22^ It is specified by:


$$\begin{array}{c} \text{logit}\left\{P(S_{\text{MGI}}=1|\text{Age},\;\text{CHD},\text{DI},\text{Race},C,\text{Smoke})\right\}=\alpha_{20}+\alpha_{21}\text{Age}+\alpha_{22}I(\text{CHD}=1)+\alpha_{23}I(\text{DI}=1)+\alpha_{24}I(\;\text{Race}=\text{NHW})+\alpha_{25}I(C=1)+\alpha_{26}I(\text{Smoke}=1)\cdot \end{array}$$

#### Auxiliary models

**AOU:** In the AOU cohort, the specific cancer subtype does not influence the selection mechanism. Therefore, we construct the auxiliary score models based on the conditional distribution of each cancer subtype given the observed covariates. The auxiliary score model for cancer subtype *c* is given by:


$$\begin{array}{cc} & P(D_{c}=1|\text{Age},\text{Sex},\text{Race},\text{HI},\text{SO},\text{IN},\text{Region},\text{Smoke},S_{\text{AOU}}=1)\\ & =P(D_{c}=1|\text{Age},\text{Sex},\text{Race},\text{HI},\text{SO},\text{IN},\text{Region},\text{Smoke})\\ & =f_{\text{AOU},c}(\text{Age},\text{Sex},\text{Race},\;\text{HI},\text{SO},\text{IN},\text{Region},\text{Smoke}), \end{array}$$

**MGI:** For auxiliary model estimation, the MGI cohort presents a more complex scenario because the specific cancer subtype is part of the selection mechanism. However, the external reference dataset lacks information on individual subtypes. To address this, we condition the auxiliary score model on the overall cancer indicator *C*, which is available in the external data. Assuming the specific subtype $D_{c}$ is independent of selection given *C* and other relevant covariates, we specify the auxiliary score model for cancer subtype *c* is given by:


$$\begin{array}{cc} & P[D_{c}=1|\text{Age},\text{CAD},\text{DI},\text{Race},C,\text{Smoke},S_{\text{MGI}}=1]\\ & =P[D_{c}=1|\text{Age},\text{CAD},\text{DI},\text{Race},C,\text{Smoke}]=f_{\text{MGI},c}(\text{Age},\text{CAD},\text{DI},\text{Race},C,\text{Smoke})\cdot \end{array}$$

### Comparison of methods and interpretation of results

**Figure 4.**
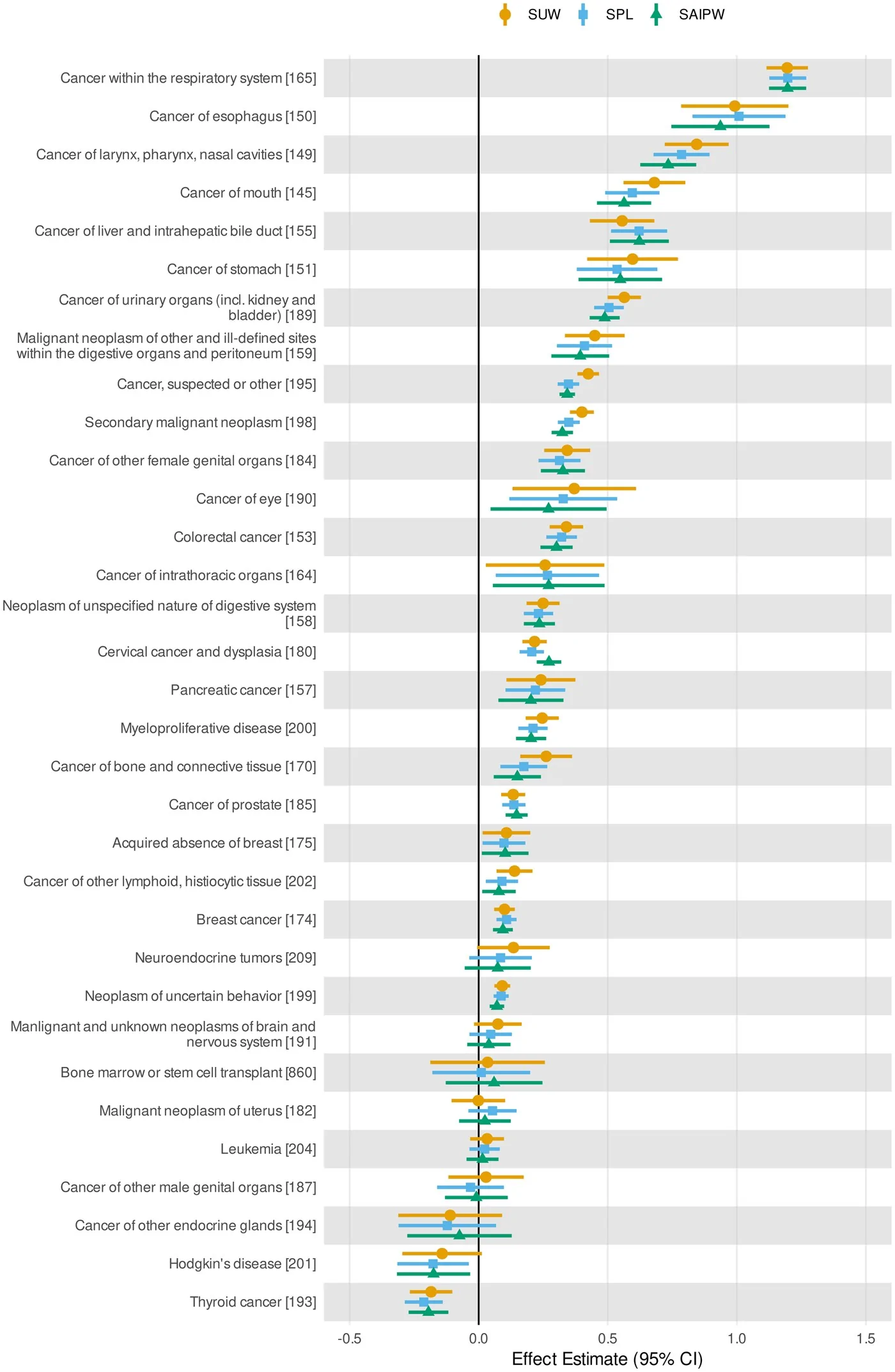
Association estimates between smoking and parent cancer types using 3 different methods: SUW, SPL, and SAIPW. Point estimates and 95% confidence intervals are shown for each method. The *x*-axis denotes cancer types and the *y*-axis represents the effect estimates. This analysis is performed using the NIH All of Us Data and MGI Anesthesiology Cohort.

In this analysis we investigated the adjusted associations between smoking and 97 distinct cancer types using SUW, SPL, and SAIPW. All models adjusted for age, sex, and race, and estimated conditional effects of smoking. Therefore, these findings may not align perfectly with marginal real-world association estimates but are intended to reflect more nuanced, adjusted associations that account for potential confounding. Figure 4 and Figure S2 illustrate the results obtained from this Cancer PheWAS association study.

Across cancer types with well-established links to smoking such as lung, laryngeal, and bladder cancers, both SPL and SAIPW methods yielded consistent positive associations. For example, phecode 145.2 (laryngeal cancer) showed strong estimates from all methods (SUW: 0.82 [0.63, 1.00], SPL: 0.84 [0.66, 1.02], SAIPW: 0.65 [0.50, 0.81]), aligning with clinical evidence that identifies smoking as a major risk factor. Similarly, for lung cancer (phecode 165.1), SAIPW estimated a log-odds of 1.22 [1.15, 1.29], consistent with known high risks associated with smoking.

**Figure 5.**
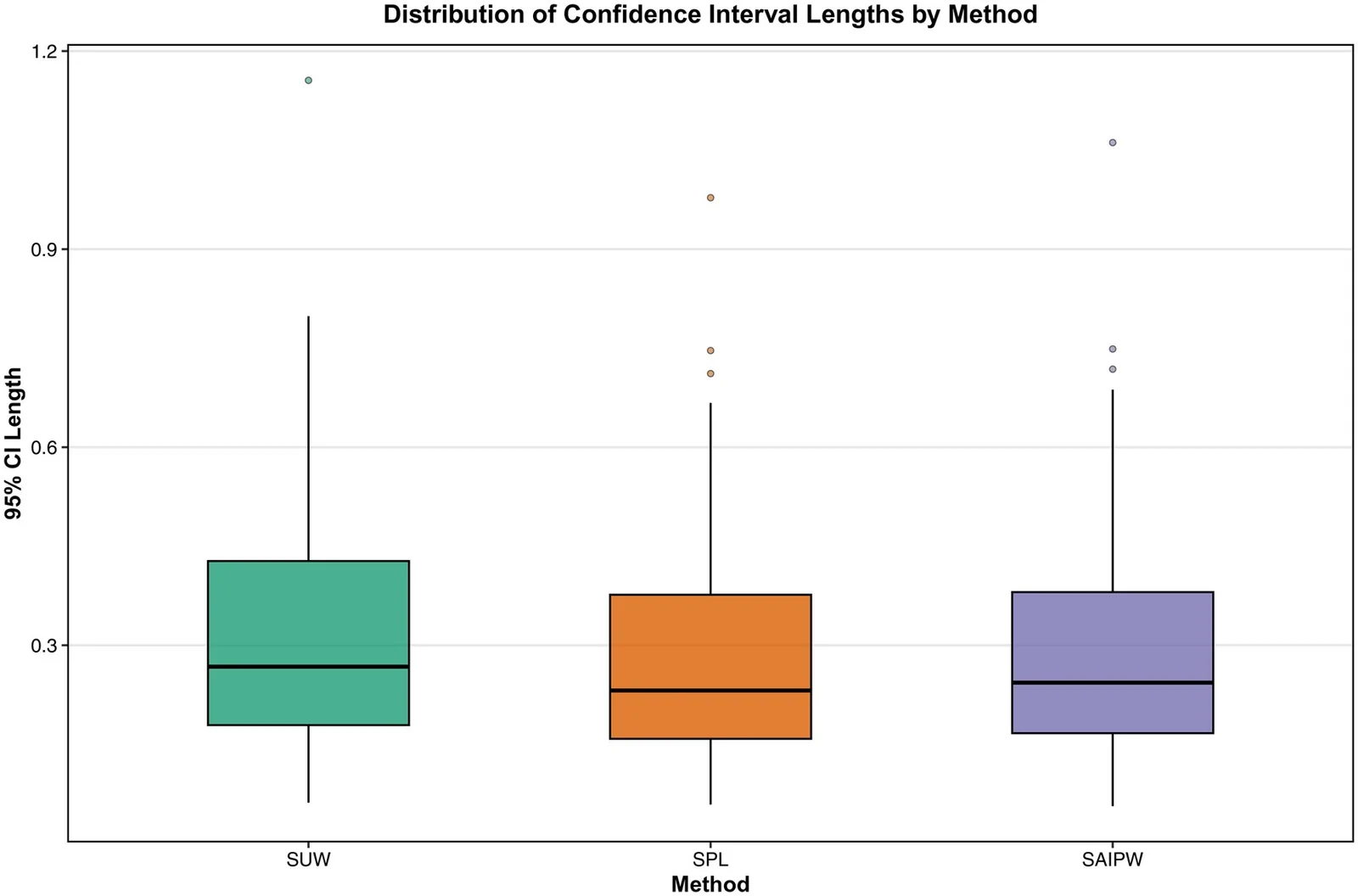
Distribution of 95% confidence interval lengths for adjusted smoking association estimates across all cancer types by method: SUW, SPL, and SAIPW. This analysis is performed using the NIH All of Us Data and MGI Anesthesiology Cohort.

**Note:** In Section S4, we performed an additional multi-site analysis of the MGI data where individual-level data could be shared across clinic sites, allowing us to implement centralized methods and confirm that our sequential framework successfully recovers these benchmark estimates without raw data sharing.

SUW often produced wider confidence intervals, particularly for rarer cancers. In contrast, SPL and SAIPW provided more stable estimates by integrating site-specific covariates with external reference data. For example, in tonsillar cancer (Phecode 149.2), SUW yielded a 95% confidence interval of [0.01, 0.75], while SPL and SAIPW produced tighter intervals of [0.11, 0.74] and [0.05, 0.68], respectively. This pattern of improved precision under SPL and SAIPW is consistent across cancer types (Figure 5), especially where prevalence is extremely low. Importantly, this improvement does not stem primarily from correcting selection bias, but rather from the fact that SPL and SAIPW borrow additional information from external sources. By leveraging such external data, these methods effectively increase the sample size for rare cancers, thereby enhancing estimation accuracy and reducing variability.

## Discussion and conclusion

This study introduces a suite of sequential methods designed to estimate association parameters in multi-site EHR research with heterogeneous sampling mechanisms under strict data-sharing constraints. We propose 2 novel estimators, SPL and SAIPW, which leverage individual-level external probability samples to correct for heterogeneous, site-specific selection biases. By restricting cross-site communication to summary-level statistics, our approach effectively enhances patient privacy while maintaining statistical rigor. Extensive simulations demonstrate that these sequential methods achieve the same statistical efficiency as centralized oracle benchmarks, providing a rigorous alternative when raw data cannot be pooled. While meta-analysis performs well in large samples, it fails in smaller sample settings due to data sparsity and shared dependencies. Notably, SAIPW offers protection against selection model misspecification through its multiply robust property. When applied to a PheWAS of 97 cancer types using AOU and MGI data, our framework consistently identified elevated risks for lung, larynx, bladder, and oral cancers, aligning with established epidemiological evidence.

Although we describe the framework as sequential, both SPL and SAIPW produce final estimates that are invariant to site order. This sequential structure is a computational mechanism designed to avoid raw data sharing, not a constraint on the analysis itself. In practice, we suggest processing sites from largest to smallest to ensure the highest degree of numerical stability.

Overall, this study provides a practical and scalable solution for valid inference in distributed health data networks. However, several considerations remain. While our framework utilizes external individual-level probability samples to mitigate selection bias, its performance remains contingent on the accuracy of these data and the corresponding sampling weights. In practice, measurement errors or missing important selection or auxiliary variables in the external source could introduce residual bias. Additionally, selection models are inherently prone to misspecification, as capturing all determinants of study participation is practically impossible in many observational settings. Therefore, our proposed IPW methods mitigate, rather than entirely eliminate, selection bias. Furthermore, the opaque nature of selection mechanisms in multi-site EHR studies makes weight estimation exceptionally challenging. While our multiply robust SAIPW approach guards against this issue to some extent via auxiliary variables, its protective benefits remain fundamentally limited if these critical covariates are unobserved.

The proposed sequential framework integrates seamlessly with modern machine learning-based nuisance estimation, supports multiply robust inference, and extends naturally to other outcome types and model classes. Future work will focus on generalizing these methods to semi-parametric and partially linear settings^23^ with relaxed assumptions for the underlying disease or outcome models, thereby enhancing their utility in real-world biomedical research. Finally, extending this work to incorporate formal privacy protections presents a promising direction for future research. Adding calibrated noise to the shared aggregate statistics could enable formal differential privacy guarantees. Additionally, integrating secure multiparty computation or homomorphic encryption, as recently described by Cho et al,^24^ could address selection bias within secure cryptographic frameworks, further eliminating residual disclosure risks.

## Supplementary Material

ocag083_Supplementary_Data

## Data Availability

Patient confidentiality prevents the sharing of data publicly. However, the data underlying the study’s results are available from the All of Us Research Program at https://www.researchallofus.org/register/ and the MGI at https://precisionhealth.umich.edu/ourresearch/michigangenomics/, for researchers who meet the criteria for confidential data access. Code for this work is available in this github link https://github.com/Ritoban1/Collaborative-Learning.
