# Supplementary material for: Privacy-enhancing sequential learning under heterogeneous selection bias in multi-site electronic health records data
Source: J Am Med Inform Assoc. 2026 Jun 16;33(9):1679–92. doi: 10.1093/jamia/ocag083 (PMC13580740; doi:10.1093/jamia/ocag083)
Supplement: Supplementary file 1 [file ocag083_supplementary_data.pdf]

Supplementary Materials: Privacy-Enhancing Sequential Learning under Heterogeneous Selection Bias in Multi-Site EHR Data

April 19, 2026

S1 Methodological Details for AIPW methods

S1.1 Centralized AIPW

We modify the centralized AIPW method of Kundu et al. (2026) to specifically address instances where an auxiliary variable $\boldsymbol{W}_j$ is unavailable in site i ($i \neq j$). This approach provides multiple robustness against selection model misspecification by combining the selection propensity model with an auxiliary score model. The auxiliary model projects the unweighted logistic score onto the space generated bty $\boldsymbol{X}_k$:

$$\mathbb{E}\left[\boldsymbol{T}(\boldsymbol{\theta}_{\boldsymbol{Z}}) \mid \boldsymbol{X}_k, S_k = 1\right] = \mathbb{E}\left[\boldsymbol{T}(\boldsymbol{\theta}_{\boldsymbol{Z}}) \mid \boldsymbol{X}_k\right] = \boldsymbol{f}_k(\boldsymbol{X}_k, \boldsymbol{\theta}_{\boldsymbol{Z}}) \cdot$$

We employ machine learning methods to estimate $\boldsymbol{f}_k(\cdot)$ by adopting a Double Machine Learning (DML) based cross fitting strategy (Chernozhukov et al., 2018) to ensure valid inference and avoid overfitting. In sites where selection is independent of D conditional on $\boldsymbol{X}_k = (\boldsymbol{Z}_{2k}, \boldsymbol{W}_k)$, we can define the auxiliary model using the conditional model, namely

$D \mid \boldsymbol{X}_k$. We use the PL method to estimate the selection propensity score. At each site $k \in \{1, 2, \ldots, K\}$, we randomly partition the available data into L mutually exclusive folds of equal size. Let the combined nuisance parameter be denoted by $\boldsymbol{\eta}_k = (\boldsymbol{\alpha}_k, \boldsymbol{f}_k)$. For each fold $l \in \{1, 2, \ldots, L\}$, we estimate the nuisance functions using the remaining $L-1$ folds, yielding $\widehat{\boldsymbol{\eta}}_k^{-l} = (\widehat{\boldsymbol{\alpha}}_k^{-l}, \widehat{\boldsymbol{f}}_k^{-l})$. Let $\mathbb{I}_{k,i}^l$ denote the indicator that i^{th} individual in the target population belongs to fold l of data from site k. The DML AIPW estimating equation for site k is then defined as:

$$\boldsymbol{U}_k^{\text{DML}}(\boldsymbol{\theta}_{\boldsymbol{Z}}, \{\widehat{\boldsymbol{\eta}}_k^{-l}\}_{l=1}^L) = \frac{1}{N} \sum_{l=1}^{L} \sum_{i=1}^{N} \frac{\mathbb{I}_{k,i}^l \cdot S_{ki}}{\pi_k(\boldsymbol{X}_{ki}, \widehat{\boldsymbol{\alpha}}_k^{-l})} \Big(\boldsymbol{T}_i(\boldsymbol{\theta}_{\boldsymbol{Z}}) - \widehat{\boldsymbol{f}}_k^{-l}(\boldsymbol{X}_{ki}, \boldsymbol{\theta}_{\boldsymbol{Z}})\Big) + \frac{1}{NL} \sum_{l=1}^{L} \sum_{i=1}^{N} \frac{S_{\text{ext},i}}{\pi_{\text{ext},i}}, \widehat{\boldsymbol{f}}_k^{-l}(\boldsymbol{X}_{ki}, \boldsymbol{\theta}_{\boldsymbol{Z}}), \tag{S1}$$

The final CAIPW estimating equation, aggregating across all sites, is then given by:

$$\sum_{k=1}^{K} U_k^{\text{DML}}(\boldsymbol{\theta}_{\boldsymbol{Z}}, \{\widehat{\boldsymbol{\eta}}_k^{-l}\}_{l=1}^L) = \mathbf{0}. \tag{S2}$$

S1.2 Sequential Augmented Inverse Probability Weighting (SAIPW)

For each site k, we define the negative gradient of AIPW estimating function from equation (S1) with respect to the target parameter as:

$$J_k^{\text{DML},\boldsymbol{\theta}_{\boldsymbol{Z}}}(\boldsymbol{\theta}_{\boldsymbol{Z}}, \{\widehat{\boldsymbol{\eta}}_k^{-l}\}_{l=1}^L) = - \left. \frac{\partial U_k^{\text{DML}}(\boldsymbol{\theta}_{\boldsymbol{Z}}, \{\widehat{\boldsymbol{\eta}}_k^{-l}\}_{l=1}^L)}{\partial \boldsymbol{\theta}_{\boldsymbol{Z}}} \right|_{\boldsymbol{\theta}_{\boldsymbol{Z}} = \boldsymbol{\theta}_{\boldsymbol{Z}}}.$$

After sequentially computing $(\widehat{\boldsymbol{\theta}}_{m,\boldsymbol{Z}}, J_m^{\text{DML},\boldsymbol{\theta}_{\boldsymbol{Z}}})$ for all $m < k$, we estimate the nuisance parameters $\{\widehat{\boldsymbol{\eta}}_k^{-l}\}_{l=1}^L$ and update $\boldsymbol{\theta}_{\boldsymbol{Z}}$ at site k by solving the following renewable estimating equation:

$$\sum_{m=1}^{k-1} J_m^{\text{DML},\boldsymbol{\theta}_{\boldsymbol{Z}}}(\widehat{\boldsymbol{\theta}}_{m,\boldsymbol{Z}}, \{\widehat{\boldsymbol{\eta}}_m^{-l}\}_{l=1}^L)(\widehat{\boldsymbol{\theta}}_{k-1,\boldsymbol{Z}} - \boldsymbol{\theta}_{\boldsymbol{Z}}) + U_k^{\text{DML}}(\boldsymbol{\theta}_{\boldsymbol{Z}}, \{\widehat{\boldsymbol{\eta}}_k^{-l}\}_{l=1}^L) = \mathbf{0}. \tag{S3}$$

S2 Variance Estimation

To facilitate variance estimation, we assume there is no patient overlap among the observed sites, although this is theoretically possible given they are sampled from the same target population. We further assume complete independence between the site data and the external individual level data. For the four primary estimators (CPL, SPL, CAIPW, and SAIPW), we evaluate variance under two extreme scenarios regarding the external data used for nuisance parameter estimation. Specifically, we consider cases where the external data samples are either completely independent across all sites or exactly identical across all sites.

S2.1 CPL

Independent External Data

The variance estimator in this case is given by,

$$\widehat{V}^{\mathrm{CPL}} = \frac{1}{N}\left(\sum_{k=1}^{K}\widehat{H}_k^{\boldsymbol{\theta}_{\boldsymbol{Z}}}\right)^{-1}\left(\sum_{k=1}^{K}\widehat{E}_k\right)\left\{\left(\sum_{k=1}^{K}\widehat{H}_k^{\boldsymbol{\theta}_{\boldsymbol{Z}}}\right)^{-1}\right\}', \tag{S4}$$

where the components are defined as follows:

$$\widehat{H}_k^{\boldsymbol{\theta}_{\boldsymbol{Z}}} = \left.\frac{\partial \boldsymbol{U}_k(\boldsymbol{\theta}_{\boldsymbol{Z}},\widehat{\boldsymbol{\alpha}}_k)}{\partial \boldsymbol{\theta}_{\boldsymbol{Z}}}\right|_{\boldsymbol{\theta}_{\boldsymbol{Z}}=\widehat{\boldsymbol{\theta}}_{k,\boldsymbol{Z}}} = -\frac{1}{N}\sum_{i=1}^{N}\frac{S_{ki}}{\pi_k(\boldsymbol{X}_{ki},\widehat{\boldsymbol{\alpha}}_k)}\left\{\frac{e^{\widehat{\boldsymbol{\theta}}'_{k,\boldsymbol{Z}}\boldsymbol{Z}_i}}{(1+e^{\widehat{\boldsymbol{\theta}}'_{k,\boldsymbol{Z}}\boldsymbol{Z}_i})^2}\right\}\boldsymbol{Z}_i\boldsymbol{Z}_i'$$

$$\widehat{E}_k = \widehat{E}_{1,k} + 2\widehat{E}_{2,k}(\widehat{M}_k^{-1'})\widehat{H}_k^{\boldsymbol{\alpha}'_k} + \widehat{H}_k^{\boldsymbol{\alpha}_k}\widehat{M}_k^{-1}\widehat{E}_{3,k}(\widehat{M}_k^{-1'})\widehat{H}_k^{\boldsymbol{\alpha}'_k} + \widehat{H}_k^{\boldsymbol{\alpha}_k}\widehat{M}_k^{-1}\widehat{E}_{\mathrm{ext},k}(\widehat{M}_k^{-1'})\widehat{H}_k^{\boldsymbol{\alpha}'_k}.$$

The individual components of $\widehat{E}_k$ are defined as:

$$\widehat{H}_k^{\boldsymbol{\alpha}_k} = \frac{\partial \boldsymbol{U}_k(\widehat{\boldsymbol{\theta}}_{k,\boldsymbol{Z}}, \boldsymbol{\alpha}_k)}{\partial \boldsymbol{\alpha}_k}\bigg|_{\boldsymbol{\alpha}_k=\widehat{\boldsymbol{\alpha}}_k} = -\frac{1}{N}\sum_{i=1}^{N} \frac{S_{ki}(1-\pi_k(\boldsymbol{X}_{ki},\widehat{\boldsymbol{\alpha}}_k))}{\pi_k(\boldsymbol{X}_{ki},\widehat{\boldsymbol{\alpha}}_k)} \left\{D_i - \frac{e^{\widehat{\boldsymbol{\theta}}'_{k,\boldsymbol{Z}}\boldsymbol{Z}_i}}{1+e^{\widehat{\boldsymbol{\theta}}'_{k,\boldsymbol{Z}}\boldsymbol{Z}_i}}\right\} \boldsymbol{Z}_i \boldsymbol{X}'_{ki},$$

$$\widehat{M}_k = \frac{\partial \boldsymbol{p}_k(\boldsymbol{\alpha}_k)}{\partial \boldsymbol{\alpha}_k}\bigg|_{\boldsymbol{\alpha}_k=\widehat{\boldsymbol{\alpha}}_k} = -\frac{1}{N}\sum_{i=1}^{N} \frac{S_{\text{ext},i}}{\pi_{\text{ext},i}} \pi_k(\boldsymbol{X}_{ki},\widehat{\boldsymbol{\alpha}}_k)(1-\pi_k(\boldsymbol{X}_{ki},\widehat{\boldsymbol{\alpha}}_k))\boldsymbol{X}_{ki}\boldsymbol{X}'_{ki},$$

$$\widehat{E}_{1,k} = \frac{1}{N}\sum_{i=1}^{N} \frac{S_{ki}}{\pi_k^2(\boldsymbol{X}_{ki},\widehat{\boldsymbol{\alpha}}_k)} \left\{D_i - \frac{e^{\widehat{\boldsymbol{\theta}}'_{k,\boldsymbol{Z}}\boldsymbol{Z}_i}}{1+e^{\widehat{\boldsymbol{\theta}}'_{k,\boldsymbol{Z}}\boldsymbol{Z}_i}}\right\}^2 \boldsymbol{Z}_i \boldsymbol{Z}'_i,$$

$$\widehat{E}_{2,k} = \frac{1}{N}\sum_{i=1}^{N} \frac{S_{ki}}{\pi_k(\boldsymbol{X}_{ki},\widehat{\boldsymbol{\alpha}}_k)} \left\{D_i - \frac{e^{\widehat{\boldsymbol{\theta}}'_{k,\boldsymbol{Z}}\boldsymbol{Z}_i}}{1+e^{\widehat{\boldsymbol{\theta}}'_{k,\boldsymbol{Z}}\boldsymbol{Z}_i}}\right\} \boldsymbol{Z}_i \boldsymbol{X}'_{ki},$$

$$\widehat{E}_{\text{ext},k} = \frac{1}{N}\sum_{i=1}^{N} \frac{S_{\text{ext},i}\pi_k^2(\boldsymbol{X}_{ki},\widehat{\boldsymbol{\alpha}}_k)}{\pi_{\text{ext},i}^2} \boldsymbol{X}_{ki}\boldsymbol{X}'_{ki}.$$

Identical External Data

The variance estimator in this case is given by,

$$\widehat{V}^{\text{CPL}} = \frac{1}{N}\left(\sum_{k=1}^{K} \widehat{H}_k^{\boldsymbol{\theta}_{\boldsymbol{Z}}}\right)^{-1} \left(\sum_{k=1}^{K} \widehat{E}_k + \widehat{E}_{\text{ext}}\right) \left\{\left(\sum_{k=1}^{K} \widehat{H}_k^{\boldsymbol{\theta}_{\boldsymbol{Z}}}\right)^{-1}\right\}', \tag{S5}$$

where the components are defined as follows:

$$\widehat{H}_k^{\boldsymbol{\theta}_{\boldsymbol{Z}}} = \frac{\partial \boldsymbol{U}_k(\boldsymbol{\theta}_{\boldsymbol{Z}}, \widehat{\boldsymbol{\alpha}}_k)}{\partial \boldsymbol{\theta}_{\boldsymbol{Z}}}\bigg|_{\boldsymbol{\theta}_{\boldsymbol{Z}}=\widehat{\boldsymbol{\theta}}_{k,\boldsymbol{Z}}} = -\frac{1}{N}\sum_{i=1}^{N} \frac{S_{ki}}{\pi_k(\boldsymbol{X}_{ki},\widehat{\boldsymbol{\alpha}}_k)} \left\{\frac{e^{\widehat{\boldsymbol{\theta}}'_{k,\boldsymbol{Z}}\boldsymbol{Z}_i}}{(1+e^{\widehat{\boldsymbol{\theta}}'_{k,\boldsymbol{Z}}\boldsymbol{Z}_i})^2}\right\} \boldsymbol{Z}_i \boldsymbol{Z}'_i$$

$$\widehat{E}_k = \widehat{E}_{1,k} + 2\widehat{E}_{2,k}(\widehat{M}_k^{-1'})\widehat{H}_k^{\boldsymbol{\alpha}'_k} + \widehat{H}_k^{\boldsymbol{\alpha}_k}\widehat{M}_k^{-1}\widehat{E}_{3,k}(\widehat{M}_k^{-1'})\widehat{H}_k^{\boldsymbol{\alpha}'_k},$$

$$\widehat{E}_{\text{ext}} = \frac{1}{N}\sum_{k=1}^{K}\sum_{l=1}^{K} \widehat{H}_k^{\boldsymbol{\alpha}_k}\widehat{M}_k^{-1}\left(\sum_{i=1}^{N} \frac{S_{\text{ext},i}\pi_k(\boldsymbol{X}_{ki},\widehat{\boldsymbol{\alpha}}_k)\pi_l(\boldsymbol{X}_{ki},\widehat{\boldsymbol{\alpha}}_l)}{\widehat{\pi}_{\text{ext},i}^2} \boldsymbol{X}_{ki}\boldsymbol{X}'_{li}\right)(\widehat{M}_l^{-1'})\widehat{H}_l^{\boldsymbol{\alpha}'_l}.$$

The individual components of $\widehat{E}_k$ are defined as:

$$\widehat{H}_k^{\boldsymbol{\alpha}_k} = \left.\frac{\partial \boldsymbol{U}_k(\widehat{\boldsymbol{\theta}}_{k,\boldsymbol{Z}}, \boldsymbol{\alpha}_k)}{\partial \boldsymbol{\alpha}_k}\right|_{\boldsymbol{\alpha}_k=\widehat{\boldsymbol{\alpha}}_k} = -\frac{1}{N}\sum_{i=1}^{N}\frac{S_{ki}(1-\pi_k(\boldsymbol{X}_{ki},\widehat{\boldsymbol{\alpha}}_k))}{\pi_k(\boldsymbol{X}_{ki},\widehat{\boldsymbol{\alpha}}_k)}\left\{D_i - \frac{e^{\widehat{\boldsymbol{\theta}}'_{k,\boldsymbol{Z}}\boldsymbol{Z}_i}}{1+e^{\widehat{\boldsymbol{\theta}}'_{k,\boldsymbol{Z}}\boldsymbol{Z}_i}}\right\}\boldsymbol{Z}_i\boldsymbol{X}'_{ki},$$

$$\widehat{M}_k = \left.\frac{\partial \boldsymbol{p}_k(\boldsymbol{\alpha}_k)}{\partial \boldsymbol{\alpha}_k}\right|_{\boldsymbol{\alpha}_k=\widehat{\boldsymbol{\alpha}}_k} = -\frac{1}{N}\sum_{i=1}^{N}\frac{S_{\text{ext},i}}{\pi_{\text{ext},i}}\pi_k(\boldsymbol{X}_{ki},\widehat{\boldsymbol{\alpha}}_k)(1-\pi_k(\boldsymbol{X}_{ki},\widehat{\boldsymbol{\alpha}}_k))\boldsymbol{X}_{ki}\boldsymbol{X}'_{ki},$$

$$\widehat{E}_{1,k} = \frac{1}{N}\sum_{i=1}^{N}\frac{S_{ki}}{\pi_k^2(\boldsymbol{X}_{ki},\widehat{\boldsymbol{\alpha}}_k)}\left\{D_i - \frac{e^{\widehat{\boldsymbol{\theta}}'_{k,\boldsymbol{Z}}\boldsymbol{Z}_i}}{1+e^{\widehat{\boldsymbol{\theta}}'_{k,\boldsymbol{Z}}\boldsymbol{Z}_i}}\right\}^2\boldsymbol{Z}_i\boldsymbol{Z}'_i,$$

$$\widehat{E}_{2,k} = \frac{1}{N}\sum_{i=1}^{N}\frac{S_{ki}}{\pi_k(\boldsymbol{X}_{ki},\widehat{\boldsymbol{\alpha}}_k)}\left\{D_i - \frac{e^{\widehat{\boldsymbol{\theta}}'_{k,\boldsymbol{Z}}\boldsymbol{Z}_i}}{1+e^{\widehat{\boldsymbol{\theta}}'_{k,\boldsymbol{Z}}\boldsymbol{Z}_i}}\right\}\boldsymbol{Z}_i\boldsymbol{X}'_{ki},$$

$$\widehat{E}_{3,k} = \frac{1}{N}\sum_{i=1}^{N}S_{ki}\boldsymbol{X}_{ki}\boldsymbol{X}'_{ki}.$$

Technically, these two scenarios differ in how the external data contribution to the E term is calculated within the variance estimators.

S2.2 SPL

We adapt the same variance formulae as CPL for the SPL estimator, justified by its $\mathcal{L}_2$-convergence to the CPL variance estimator. Implementation is however performed sequentially.

Independent External Data

At the first site, we evaluate the components $\widehat{H}_1^{\boldsymbol{\theta}_{\boldsymbol{Z}}}$ and $\widehat{E}_1$ using the parameter estimates $\widehat{\boldsymbol{\theta}}_{1,\boldsymbol{Z}}$ and $\widehat{\boldsymbol{\alpha}}_1$. Because $\widehat{\boldsymbol{\theta}}_{1,\boldsymbol{Z}}$ and the gradient component are already transmitted for the point estimation procedure, $\widehat{E}_1$ represents the only additional term transmitted specifically for variance estimation. Consequently, for any intermediate site $1 \leq k < K$, we compute the cumulative sums $\left(\sum_{l=1}^{k}\widehat{H}_l^{\boldsymbol{\theta}_{\boldsymbol{Z}}}\right)$ and $\left(\sum_{l=1}^{k}\widehat{E}_l\right)$ and transmit them to the $(k+1)^{\text{th}}$ site alongside the updated parameter estimate $\widehat{\boldsymbol{\theta}}_{k,\boldsymbol{Z}}$. Through this sequential aggregation, we obtain the final variance formulation at the terminal K^{th} site, as specified in Equation (S4).

Identical External Data

In this case, the sequential computation of the variance is more computationally involved due to the complex structure of $\widehat{E}_{\text{ext}}$. To optimize this procedure, we utilize an algebraic factorization. Let n_{ext} denote the total number of individuals in the external probability sample. For each site $k \in \{1, 2, \dots, K\}$ and each external subject $i \in \{1, 2, \dots, n_{\text{ext}}\}$, we define the site specific vector $\boldsymbol{\psi}_{ki} = \widehat{H}_k^{\boldsymbol{\alpha}_k} \widehat{M}_k^{-1} \pi_k(\boldsymbol{X}_{ki}, \widehat{\boldsymbol{\alpha}}_k) \boldsymbol{X}_{ki}$.

At the first site, we compute $\widehat{H}_1^{\boldsymbol{\theta}_{\boldsymbol{Z}}}$, $\widehat{E}_1$, and the initial external data vectors $\boldsymbol{\psi}_{1i}$ for all n_{ext} subjects using the parameter estimates $\widehat{\boldsymbol{\theta}}_{1,\boldsymbol{Z}}$ and $\widehat{\boldsymbol{\alpha}}_1$. Consequently, for any intermediate site $1 \leq k < K$, we compute the cumulative matrix sums $\left(\sum_{l=1}^{k} \widehat{H}_l^{\boldsymbol{\theta}_{\boldsymbol{Z}}}\right)$ and $\left(\sum_{l=1}^{k} \widehat{E}_l\right)$. Crucially, instead of retaining the full history of individual vectors, we compute the cumulative running sum for each external subject as $\boldsymbol{V}_{ki} = \sum_{l=1}^{k} \boldsymbol{\psi}_{li}$. These aggregated statistics are then transmitted to the $(k+1)^{\text{th}}$ site alongside the updated parameter estimate $\widehat{\boldsymbol{\theta}}_{k,\boldsymbol{Z}}$. Finally, at the terminal K^{th} site, we compute the ultimate cumulative sums. Using the final aggregated vectors $\boldsymbol{V}_{Ki} = \sum_{k=1}^{K} \boldsymbol{\psi}_{ki}$, the external data correction term can be evaluated efficiently in a single pass as:

$$\widehat{E}_{\text{ext}} = \frac{1}{N} \sum_{i=1}^{N} \frac{S_{\text{ext},i}}{\pi_{\text{ext},i}^2} \boldsymbol{V}_{Ki} \boldsymbol{V}'_{Ki}$$

By integrating this computed term with the other shared statistics, we obtain the complete variance formulation specified by Equation (S5).

Privacy Implications for Variance Estimation: To appropriately adjust for selection bias, our method requires transmitting the additional cumulative matrices $\left(\sum_{l=1}^{k} \widehat{E}_l\right)$ compared to the unweighted baseline. While this necessitates sharing a greater volume of summary statistics, the complex, composite mathematical structure of these variance components serves to mask individual contributions, making it highly difficult to reverse engineer the original patient data. Furthermore, the variance terms associated with the external probability sample

are computed strictly from data for which individual level records are already accessible to all participating sites. Consequently, sharing these specific external components is unlikely to introduce substantive additional disclosure risk regarding the private clinical populations.

S2.3 CAIPW

Under correct specification of both the selection model $\pi_k(\cdot)$ and the auxiliary score model $\boldsymbol{f}_k(\cdot)$ for each site $k \in \{1, 2, \ldots, K\}$, the influence function corresponding to the estimating equation (S5) satisfies the Neyman orthogonality property. This property allows us to ignore the contribution of nuisance parameter estimation to the variance, thereby simplifying variance computation.

Independent External Data

The variance estimator in this case is given by,

$$\widehat{V}_{\text{AIPW}}^{\text{DML}} = \frac{1}{N}\left(\sum_{k=1}^{K}\widehat{H}_k^{\text{DML},\boldsymbol{\theta_Z}}\right)^{-1}\left(\sum_{k=1}^{K}\widehat{E}_k^{\text{DML}}\right)\left\{\left(\sum_{k=1}^{K}\widehat{H}_k^{\text{DML},\boldsymbol{\theta_Z}}\right)^{-1}\right\}', \tag{S6}$$

where the components are defined as follows:

$$\begin{aligned}
\widehat{H}_k^{\text{DML},\boldsymbol{\theta_Z}} &= \left.\frac{\partial \boldsymbol{U}_k^{\text{DML}}(\boldsymbol{\theta_Z}, \{\widehat{\boldsymbol{\eta}}_k^{-l}\}_{l=1}^{L})}{\partial \boldsymbol{\theta_Z}}\right|_{\boldsymbol{\theta_Z}=\widehat{\boldsymbol{\theta}}_{k,\boldsymbol{Z}}} \\
&= \frac{1}{N}\sum_{l=1}^{L}\sum_{i=1}^{N}\frac{S_{ki}\mathbb{I}_{k,i}^{l}}{\pi_k(\boldsymbol{X}_{ki}, \widehat{\boldsymbol{\alpha}}_k^{-l})}\left(\left\{-\frac{e^{\widehat{\boldsymbol{\theta}}_{k,\boldsymbol{Z}}'\boldsymbol{Z}_i}}{(1+e^{\widehat{\boldsymbol{\theta}}_{k,\boldsymbol{Z}}'\boldsymbol{Z}_i})^2}\right\}\boldsymbol{Z}_i\boldsymbol{Z}_i' - \left.\frac{\partial \widehat{\boldsymbol{f}}_k^{-l}(\boldsymbol{X}_{ki}, \boldsymbol{\theta_Z})}{\partial \boldsymbol{\theta_Z}}\right|_{\boldsymbol{\theta_Z}=\widehat{\boldsymbol{\theta}}_{k,\boldsymbol{Z}}}\right) \\
&+ \frac{1}{NL}\sum_{l=1}^{L}\sum_{i=1}^{N}\frac{S_{\text{ext},i}}{\pi_{\text{ext},i}}\left.\frac{\partial \widehat{\boldsymbol{f}}_k^{-l}(\boldsymbol{X}_{ki}, \boldsymbol{\theta_Z})}{\partial \boldsymbol{\theta_Z}}\right|_{\boldsymbol{\theta_Z}=\widehat{\boldsymbol{\theta}}_{k,\boldsymbol{Z}}} \\
\widehat{E}_k^{\text{DML}} &= \widehat{E}_{1k}^{\text{DML}} + \widehat{E}_{\text{ext},k}^{\text{DML}}
\end{aligned}$$

The individual components of $\widehat{E}_k^{\text{DML}}$ are defined as follows:

$$\widehat{E}_{1k}^{\text{DML}} = \frac{1}{N}\sum_{l=1}^{L}\sum_{i=1}^{N}\frac{\mathbb{I}_{k,i}^{l}\cdot S_{ki}}{\pi_k^2(\boldsymbol{X}_{ki},\widehat{\boldsymbol{\alpha}}_k^{-l})}\left(\boldsymbol{T}_i(\widehat{\boldsymbol{\theta}}_{k,\boldsymbol{Z}})-\widehat{\boldsymbol{f}}_k^{-l}(\boldsymbol{X}_{ki},\widehat{\boldsymbol{\theta}}_{k,\boldsymbol{Z}})\right)\left(\boldsymbol{T}_i(\widehat{\boldsymbol{\theta}}_{k,\boldsymbol{Z}})-\widehat{\boldsymbol{f}}_k^{-l}(\boldsymbol{X}_{ki},\widehat{\boldsymbol{\theta}}_{k,\boldsymbol{Z}})\right)'$$

$$\widehat{E}_{\text{ext},k}^{\text{DML}} = \frac{1}{N}\sum_{i=1}^{N}\frac{S_{\text{ext},i}}{\pi_{\text{ext},i}^2}\left[\left(\sum_{l=1}^{L}\frac{\widehat{\boldsymbol{f}}_k^{-l}(\boldsymbol{X}_{ki},\widehat{\boldsymbol{\theta}}_{k,\boldsymbol{Z}})}{L}\right)\left(\sum_{l=1}^{L}\frac{\widehat{\boldsymbol{f}}_k^{-l}(\boldsymbol{X}_{ki},\widehat{\boldsymbol{\theta}}_{k,\boldsymbol{Z}})}{L}\right)'\right]$$

Identical External Data

The variance estimator in this case is given by,

$$\widehat{V}_{\text{AIPW}}^{\text{DML}} = \frac{1}{N}\left(\sum_{k=1}^{K}\widehat{H}_k^{\text{DML},\boldsymbol{\theta}_{\boldsymbol{Z}}}\right)^{-1}\left(\sum_{k=1}^{K}\widehat{E}_k^{\text{DML}}+\widehat{E}_{\text{ext}}^{\text{DML}}\right)\left\{\left(\sum_{k=1}^{K}\widehat{H}_k^{\text{DML},\boldsymbol{\theta}_{\boldsymbol{Z}}}\right)^{-1}\right\}', \tag{S7}$$

where the components are defined as follows:

$$\widehat{H}_k^{\text{DML},\boldsymbol{\theta}_{\boldsymbol{Z}}} = \left.\frac{\partial \boldsymbol{U}_k^{\text{DML}}(\boldsymbol{\theta}_{\boldsymbol{Z}},\{\widehat{\boldsymbol{\eta}}_k^{-l}\}_{l=1}^{L})}{\partial\boldsymbol{\theta}_{\boldsymbol{Z}}}\right|_{\boldsymbol{\theta}_{\boldsymbol{Z}}=\widehat{\boldsymbol{\theta}}_{k,\boldsymbol{Z}}}$$

$$= \frac{1}{N}\sum_{l=1}^{L}\sum_{i=1}^{N}\frac{S_{ki}\mathbb{I}_{k,i}^{l}}{\pi_k(\boldsymbol{X}_{ki},\widehat{\boldsymbol{\alpha}}_k^{-l})}\left(\left\{-\frac{e^{\widehat{\boldsymbol{\theta}}_{k,\boldsymbol{Z}}'\boldsymbol{Z}_i}}{(1+e^{\widehat{\boldsymbol{\theta}}_{k,\boldsymbol{Z}}'\boldsymbol{Z}_i})^2}\right\}\boldsymbol{Z}_i\boldsymbol{Z}_i' - \left.\frac{\partial\widehat{\boldsymbol{f}}_k^{-l}(\boldsymbol{X}_{ki},\boldsymbol{\theta}_{\boldsymbol{Z}})}{\partial\boldsymbol{\theta}_{\boldsymbol{Z}}}\right|_{\boldsymbol{\theta}_{\boldsymbol{Z}}=\widehat{\boldsymbol{\theta}}_{k,\boldsymbol{Z}}}\right)$$

$$+\frac{1}{NL}\sum_{l=1}^{L}\sum_{i=1}^{N}\frac{S_{\text{ext},i}}{\pi_{\text{ext},i}}\left.\frac{\partial\widehat{\boldsymbol{f}}_k^{-l}(\boldsymbol{X}_{ki},\boldsymbol{\theta}_{\boldsymbol{Z}})}{\partial\boldsymbol{\theta}_{\boldsymbol{Z}}}\right|_{\boldsymbol{\theta}_{\boldsymbol{Z}}=\widehat{\boldsymbol{\theta}}_{k,\boldsymbol{Z}}}$$

$$\widehat{E}_{k}^{\text{DML}} = \frac{1}{N}\sum_{l=1}^{L}\sum_{i=1}^{N}\frac{\mathbb{I}_{k,i}^{l}\cdot S_{ki}}{\pi_k^2(\boldsymbol{X}_{ki},\widehat{\boldsymbol{\alpha}}_k^{-l})}\left(\boldsymbol{T}_i(\widehat{\boldsymbol{\theta}}_{k,\boldsymbol{Z}})-\widehat{\boldsymbol{f}}_k^{-l}(\boldsymbol{X}_{ki},\widehat{\boldsymbol{\theta}}_{k,\boldsymbol{Z}})\right)\left(\boldsymbol{T}_i(\widehat{\boldsymbol{\theta}}_{k,\boldsymbol{Z}})-\widehat{\boldsymbol{f}}_k^{-l}(\boldsymbol{X}_{ki},\widehat{\boldsymbol{\theta}}_{k,\boldsymbol{Z}})\right)'$$

$$\widehat{E}_{\text{ext}}^{\text{DML}} = \frac{1}{N}\sum_{i=1}^{N}\frac{S_{\text{ext},i}}{\pi_{\text{ext},i}^2}\left[\left(\sum_{k=1}^{K}\sum_{l=1}^{L}\frac{\widehat{\boldsymbol{f}}_k^{-l}(\boldsymbol{X}_{ki},\widehat{\boldsymbol{\theta}}_{k,\boldsymbol{Z}})}{L}\right)\left(\sum_{k=1}^{K}\sum_{l=1}^{L}\frac{\widehat{\boldsymbol{f}}_k^{-l}(\boldsymbol{X}_{ki},\widehat{\boldsymbol{\theta}}_{k,\boldsymbol{Z}})}{L}\right)'\right]$$

S2.4 SAIPW

For SAIPW, we use the same variance formulae as CAIPW, but we compute it sequentially.

Independent External Data

At the first site, we evaluate the components $\widehat{H}_1^{\mathrm{DML},\boldsymbol{\theta}_{\boldsymbol{Z}}}$ and $\widehat{E}_1^{\mathrm{DML}}$. Because the parameter estimate $\widehat{\boldsymbol{\theta}}_{1,\boldsymbol{Z}}$ and the gradient component are inherently transmitted during the primary point estimation procedure, $\widehat{E}_1^{\mathrm{DML}}$ constitutes the sole supplementary term transmitted specifically for variance calculation. Consequently, for any intermediate site $1 \leq k < K$, we compute the cumulative sums $\left(\sum_{l=1}^{k} \widehat{H}_l^{\mathrm{DML},\boldsymbol{\theta}_{\boldsymbol{Z}}}\right)$ and $\left(\sum_{l=1}^{k} \widehat{E}_l^{\mathrm{DML}}\right)$ and transmit them to the $(k+1)^{\mathrm{th}}$ site alongside the updated parameter estimate $\widehat{\boldsymbol{\theta}}_{k,\boldsymbol{Z}}$. Through this sequential aggregation, we obtain the complete variance formulation at the terminal K^{th} site, as specified in Equation (S6).

Identical External Data

In this case, the sequential computation of the variance is more computationally involved due to the complex structure of $\widehat{E}_{\mathrm{ext}}^{\mathrm{DML}}$. For each site $k \in \{1, 2, \ldots, K\}$ and each external subject $i \in \{1, 2, \ldots, n_{\mathrm{ext}}\}$, we define the site specific vector $\boldsymbol{\psi}_{ki} = \frac{1}{L}\sum_{l=1}^{L} \widehat{\boldsymbol{f}}_k^{-l}(\boldsymbol{X}_{ki}, \widehat{\boldsymbol{\theta}}_{k,\boldsymbol{Z}})$.

At the first site, we compute $\widehat{H}_1^{\mathrm{DML},\boldsymbol{\theta}_{\boldsymbol{Z}}}$, $\widehat{E}_1^{\mathrm{DML}}$, and the initial external data vectors $\boldsymbol{\psi}_{1i}$ for all n_{ext} subjects. Consequently, for any intermediate site $1 \leq k < K$, we compute the cumulative matrix sums $\left(\sum_{m=1}^{k} \widehat{H}_m^{\mathrm{DML},\boldsymbol{\theta}_{m,\boldsymbol{Z}}}\right)$ and $\left(\sum_{m=1}^{k} \widehat{E}_m^{\mathrm{DML}}\right)$. Crucially, instead of retaining the full history of individual vectors, we compute the cumulative running sum for each external subject as $\boldsymbol{V}_{ki} = \sum_{m=1}^{k} \boldsymbol{\psi}_{mi}$. These aggregated statistics are then transmitted to the $(k+1)^{\mathrm{th}}$ site alongside the updated parameter estimate $\widehat{\boldsymbol{\theta}}_{k,\boldsymbol{Z}}$. Finally, at the terminal K^{th} site, we compute the ultimate cumulative sums. Using the final aggregated vectors $\boldsymbol{V}_{Ki} = \sum_{m=1}^{K} \boldsymbol{\psi}_{mi}$, the external data correction term can be evaluated efficiently in a single pass as:

$$\widehat{E}_{\mathrm{ext}}^{\mathrm{DML}} = \frac{1}{N}\sum_{i=1}^{N} \frac{S_{\mathrm{ext},i}}{\pi_{\mathrm{ext},i}^2} \boldsymbol{V}_{Ki}\boldsymbol{V}'_{Ki}$$

By integrating this computed term with the other shared statistics, we obtain the complete

variance formulation specified by Equation (S7).

Privacy Implications for Variance Estimation: Similar to SPL, calculating the SAIPW variance requires transmitting additional cumulative matrices $\left(\sum_{l=1}^{k} \widehat{E}_l\right)$. However, the reliance on machine learning auxiliary models renders these aggregate terms analytically more opaque. This advanced algorithmic complexity affords proportionally greater shielding of individual data points, creating a substantially higher barrier against the reverse engineering of patient level information.

Communication cost for Variance Estimation: For variance estimation, the communication cost depends heavily on the structure of the external data. When external samples are independent across sites, sharing the additional cumulative variance matrices $\left(\sum_{l=1}^{k} \widehat{E}_l\right)$ incurs an additional transmission cost of strictly $O(p^2)$. Conversely, when external samples are identical across sites, the algorithm must also transmit the cumulative external data vectors $\boldsymbol{V}_{ki}$. This necessitates transferring an $n_{\text{ext}} \times (p+1)$ matrix, resulting in a total variance communication complexity of $O(p^2) + O(n_{\text{ext}} \cdot p)$. Although the external probability sample size n_{ext} is typically smaller than the primary study population, this elevated transmission burden remains a practical limitation for the identical data scenario, particularly if the external dataset is massive. These communication scaling laws and their associated limitations apply equally to both the SPL and SAIPW frameworks.

S3 Cancer Phenotypes List

In this study, phenotypes are defined using the PheWAS R package (Version 0.99.5-4), which maps ICD-9-CM and ICD-10-CM codes to PheWAS codes (PheCodes), resulting in up to 1,817 distinct codes. For our analysis, the PheCodes for cancer include: 145, 145.1, 145.2, 145.3, 145.4, 145.5, 149, 149.1, 149.2, 149.3, 149.4, 149.5, 149.9, 150, 151, 153, 153.2, 153.3,

155, 155.1, 157, 158, 159, 159.2, 159.3, 159.4, 164, 165, 165.1, 170, 170.1, 170.2, 172.1, 172.11, 174, 174.1, 174.11, 174.2, 174.3, 175, 180, 180.1, 180.3, 182, 184, 184.1, 184.11, 184.2, 185, 187, 187.1, 187.2, 187.8, 189, 189.1, 189.11, 189.12, 189.2, 189.21, 189.4, 190, 191, 191.1, 191.11, 193, 194, 195, 195.1, 195.3, 196, 197, 198, 198.1, 198.2, 198.3, 198.4, 198.5, 198.6, 198.7, 199, 199.4, 200, 200.1, 201, 202, 202.2, 202.21, 202.22, 202.23, 202.24, 204, 204.1, 204.11, 204.12, 204.2, 204.21, 204.22, 204.3, 204.4, 209, and 860.

S4 MGI Multi Site Data Analysis

Setup: In the primary data analysis presented in the main text, we considered only the largest single clinic within the Michigan Genomics Initiative (MGI). However, since 2012, MGI has recruited individuals through at least six major specialized clinics: MGI Anesthesiology (BB), MIPACT (Michigan Predictive Activity and Clinical Trajectories), MEND (Metabolism, Endocrinology, and Diabetes), MHB (Mental Health BioBank), PROMPT (PROviding Mental health Precision Treatment), and MY PART (Michigan and You – Partnering to Advance Research Together, a diverse cohort oversampling underrepresented populations). This clinic-based recruitment strategy is common in hospital-based biobanking, but it naturally gives rise to different participant profiles across sub-cohorts as a result of heterogeneous, site-specific patient selection mechanisms.

Analysis: This supplementary analysis aims to evaluate the performance of our proposed sequential methods against centralized methods in a multi-site setting. To do this, we treat distinct MGI clinics as separate data sites. While data across these MGI sites can technically be pooled, we apply our sequential methods under the operational constraint that only summary-level data can be shared, comparing the results to the centralized oracle approach. We utilized data from three specific cohorts: MGI Anesthesiology (BB) ($n = 50,935$), MIPACT ($n = 6,056$), and MEND ($n = 2,527$). The relatively small sample sizes of MIPACT

(a notably healthier cohort) and MEND present challenges for studying rarer cancer outcomes (PheWAS codes). Furthermore, implementing both the DML AIPW estimators (CAIPW, SAIPW) in this setting is practically infeasible; the requisite cross-fitting for complex machine learning models often results in data folds with zero or extremely few cases, leading to highly unstable estimates. Consequently, we restricted this analysis to the unweighted methods (CUW, SUW) and the IPW-based pseudo-likelihood methods (CPL, SPL). To ensure model stability, we excluded cancer types where the prevalence in either MEND or MIPACT was effectively zero, leaving 78 out of 100 original cancer codes for the final analysis.

Result: Figures S3 and S4 provide a comprehensive visual summary of the results for the unweighted (UW) methods, detailing the adjusted smoking association estimates for both parent and child cancer codes. In parallel, Figures S5 and S6 present the corresponding association estimates derived from the pseudo-likelihood (PL) methods. A key observation across all evaluated cancer outcomes is that the proposed sequential methods (SUW and SPL) perform highly comparable to their centralized, oracle counterparts (CUW and CPL). Ultimately, this strong empirical alignment confirms that our sequential framework successfully and reliably recovers the centralized effect estimates, fully circumventing the need for individual-level data pooling while strictly adhering to privacy constraints.

References

V. Chernozhukov, D. Chetverikov, M. Demirer, E. Duflo, C. Hansen, W. Newey, and J. Robins. Double/debiased machine learning for treatment and structural parameters, 2018.

R. Kundu, X. Shi, M. Kleinsasser, L. Fritsche, M. Salvatore, and B. Mukherjee. A doubly robust framework for addressing outcome-dependent selection bias in multi-cohort ehr studies. *Biostatistics*, 27(1):kxag001, 2026.

**Abbr.**	**Definition**	**Abbr.**	**Definition**	**Abbr.**	**Definition**
**AIPW**	Augmented Inverse Probability Weighting	**GBMI**	Global Biobank Meta-analysis	**NHW**	Non-Hispanic White
**AOU**	All of Us	**HI**	Health Insurance	**NIH**	National Institutes of Health
**BMI**	Body Mass Index	**HIPAA**	Health Insurance Portability and Accountability Act	**PheWAS**	Phenome-Wide Association Study
**C**	Cancer	**HITECH**	Health Information Technology for Economic and Clinical Health	**RBP**	Relative Bias Percentage
**CAIPW**	Centralized Augmented IPW	**IPW**	Inverse Probability Weighting	**RE**	Relative Efficiency
**CHD**	Coronary Heart Disease	**MAIPW**	Meta Augmented IPW	**RMSE**	Relative Mean Squared Error
**CP**	Coverage Probability	**MGI**	Michigan Genomics Initiative	**SAIPW**	Sequential Augmented IPW
**CPL**	Centralized Pseudo-Likelihood	**MPL**	Meta Pseudo-Likelihood	**SO**	Sexual Orientation
**CUW**	Centralized Unweighted	**MUW**	Meta Unweighted	**SPL**	Sequential Pseudo-Likelihood
**DAG**	Directed Acyclic Graph	**MVP**	Million Veteran Program	**SUW**	Sequential Unweighted
**DI**	Type 2 Diabetes	**NHIS**	National Health Interview Survey		
**DML**	Double Machine Learning	**EHR**	Electronic Health Records		

Table S1: List of Abbreviations

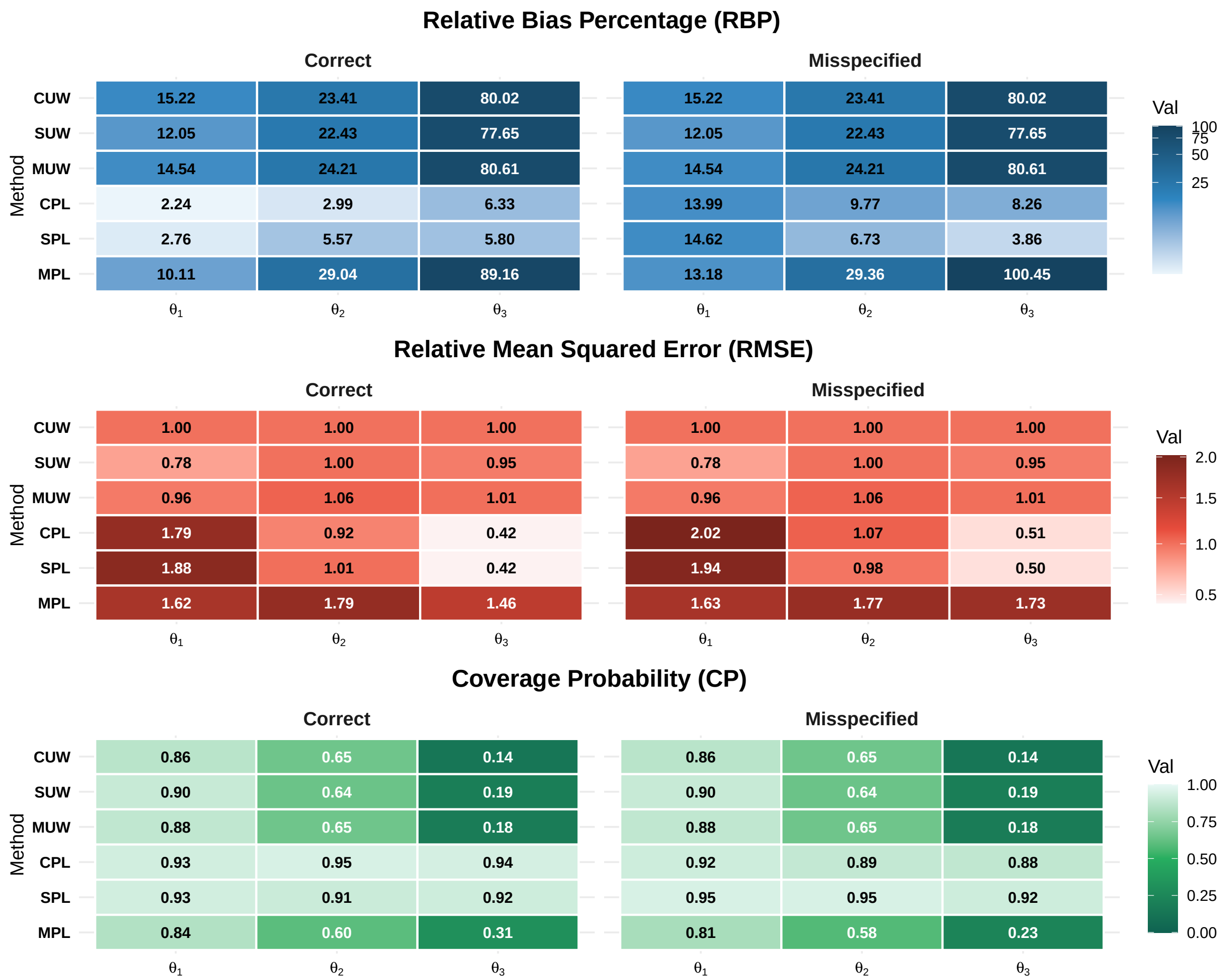

Figure S1: Performance metrics of unweighted, centralized, sequential, and meta-analysis estimators across two selection model specification scenarios under a setup with large number of sites ($K = 20$). Results are presented for Relative Bias Percentage (RBP), Relative Mean Squared Error (RMSE), and Coverage Probability (CP) for target parameters θ_1, θ_2, and θ_3. The scenarios include a correctly specified model and a heterogeneous misspecification scenario.

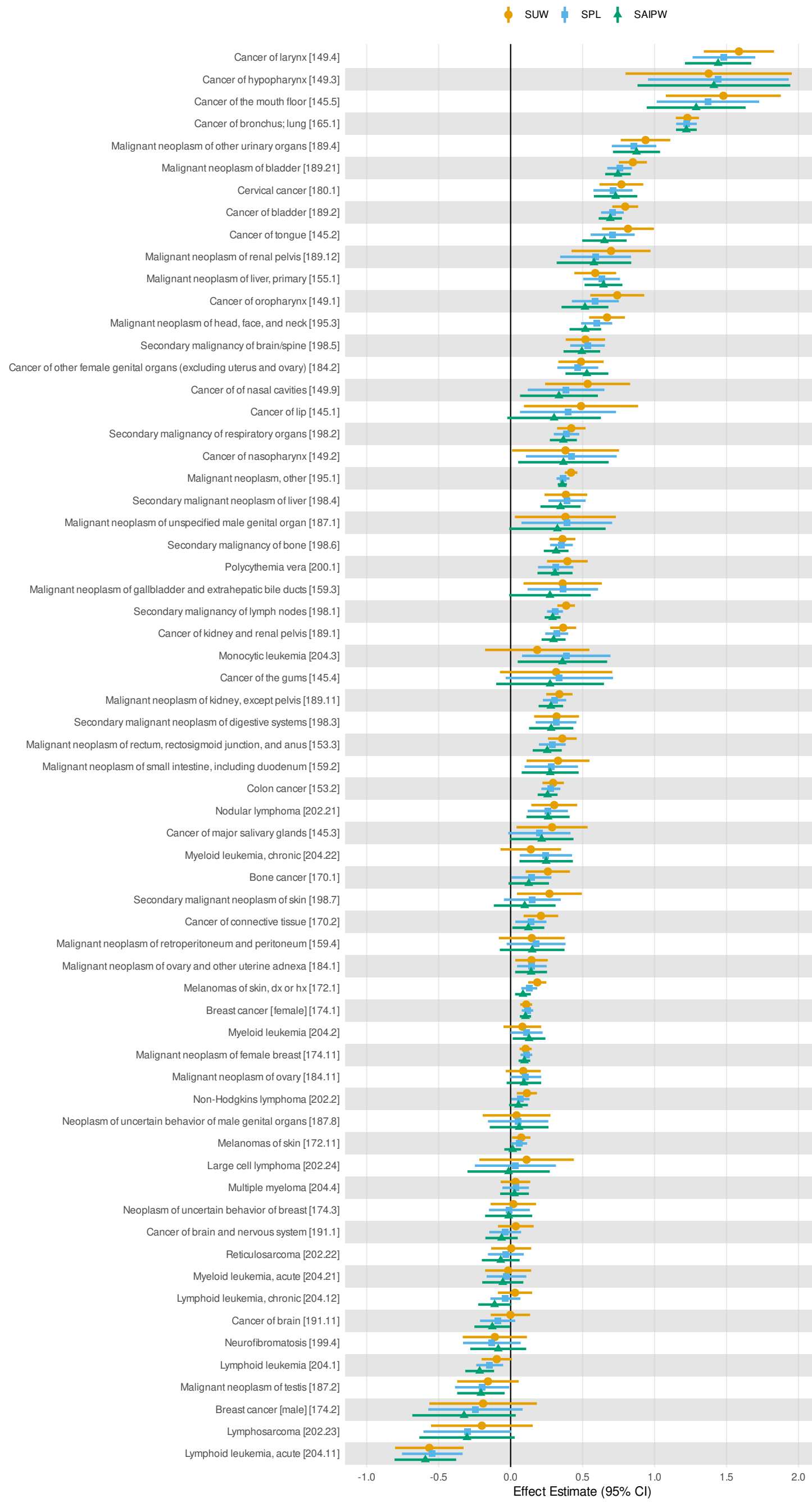

Figure S2: Association estimates between smoking and the children cancer types using three different methods: SUW (Sequential Unweighted), SPL (Sequential Pseudo-Likelihood), and SAIPW (Sequential Augmented Inverse Probability Weighting). Point estimates and 95% confidence intervals are shown for each method. The x-axis denotes cancer types and the y-axis represents the effect estimates. This analysis is performed using the NIH All of Us Data and MGI Anesthesiology Cohort.

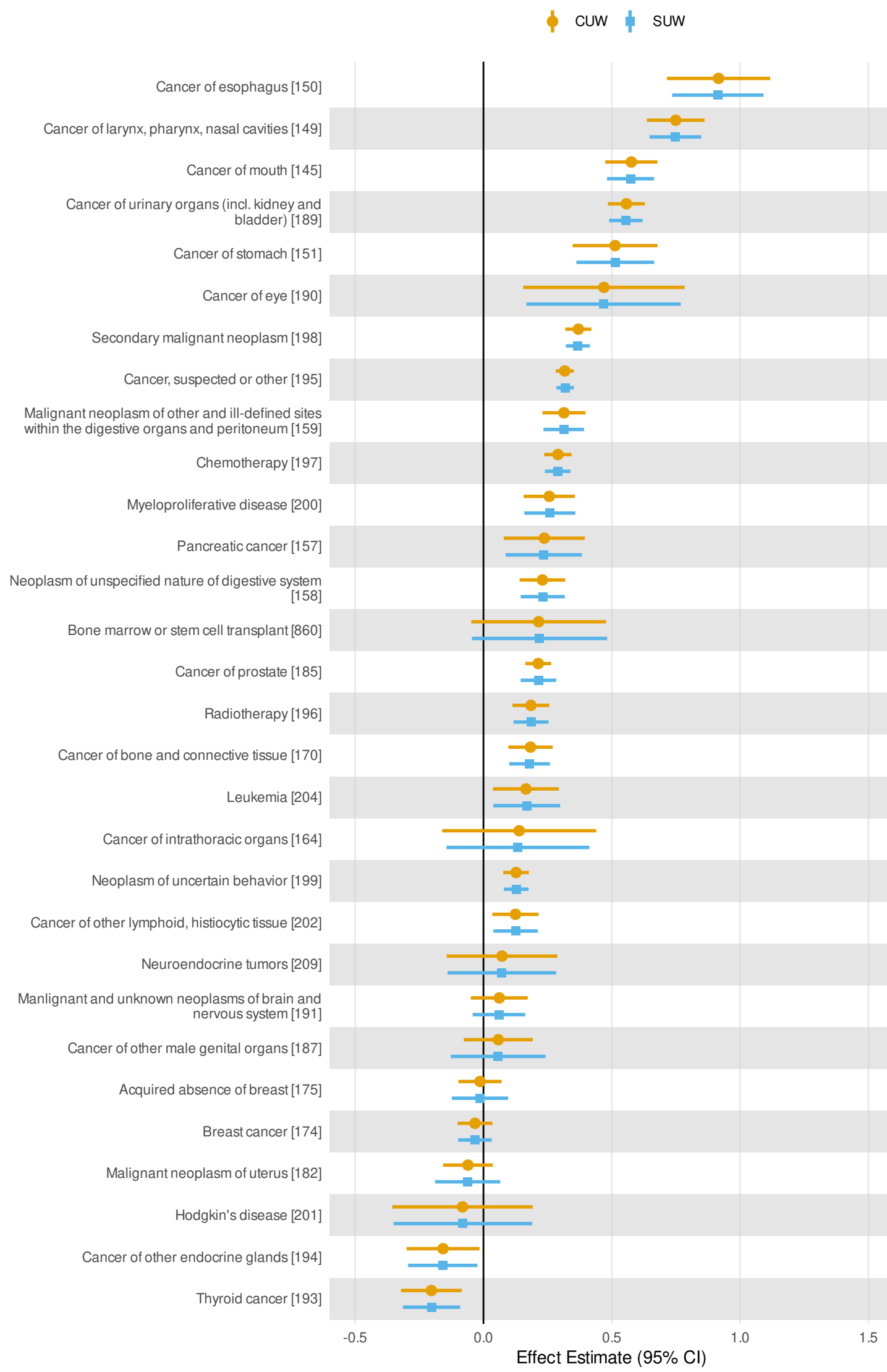

Figure S3: Association estimates between smoking and parent cancer types using two unweighted methods: CUW (Centralized Unweighted) and SUW (Sequential Unweighted). Point estimates and 95% confidence intervals are shown for each method. The y-axis denotes cancer types and the x-axis represents the effect estimates. This analysis is performed using the multi-site MGI cohort, comprising the Anesthesiology, MIPACT, and MEND clinics.

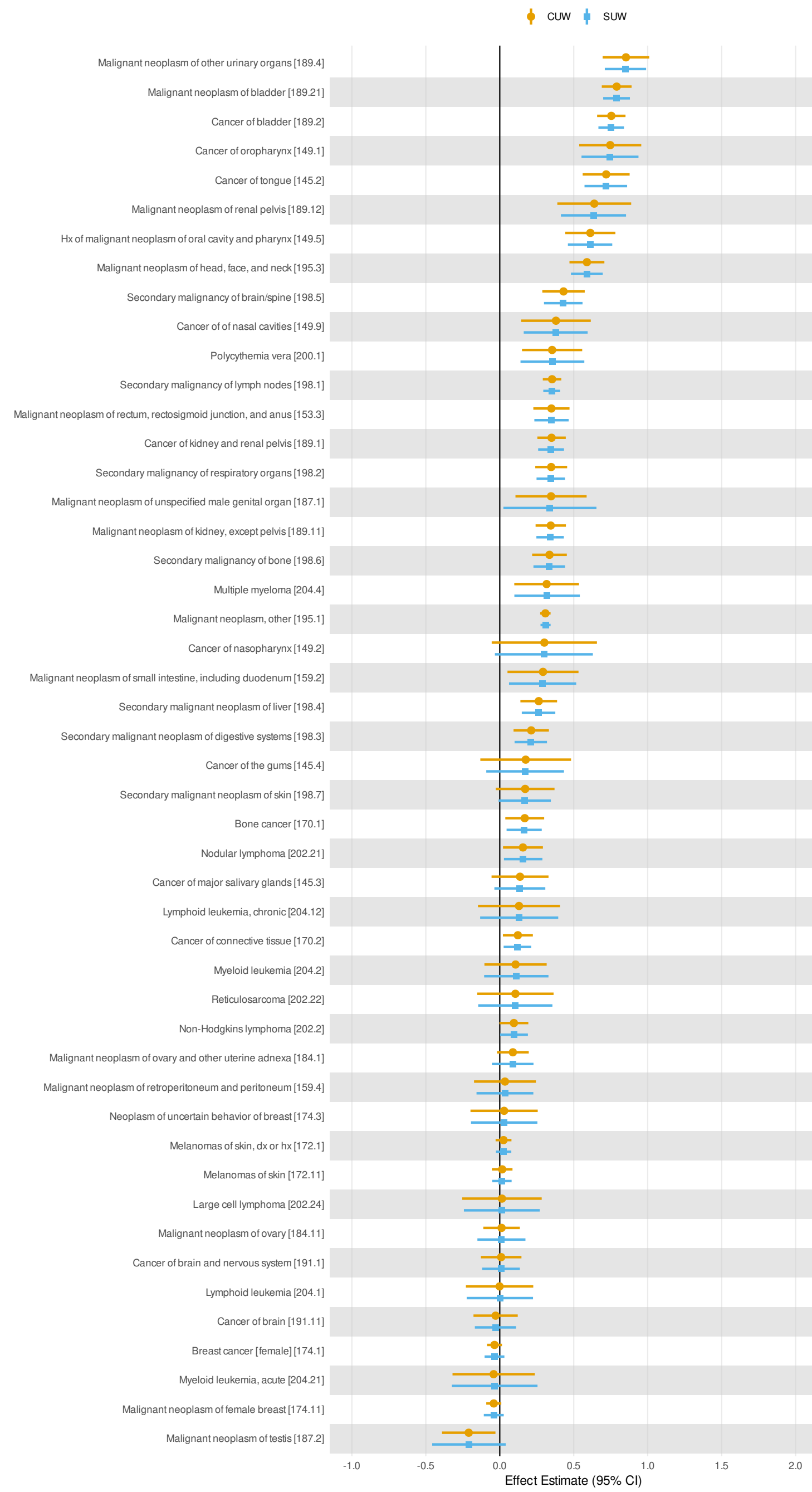

Figure S4: Association estimates between smoking and children cancer types using two unweighted methods: CUW (Centralized Unweighted) and SUW (Sequential Unweighted). Point estimates and 95% confidence intervals are shown for each method. The y-axis denotes cancer types and the x-axis represents the effect estimates. This analysis is performed using the multi-site MGI cohort, comprising the Anesthesiology, MIPACT, and MEND clinics.

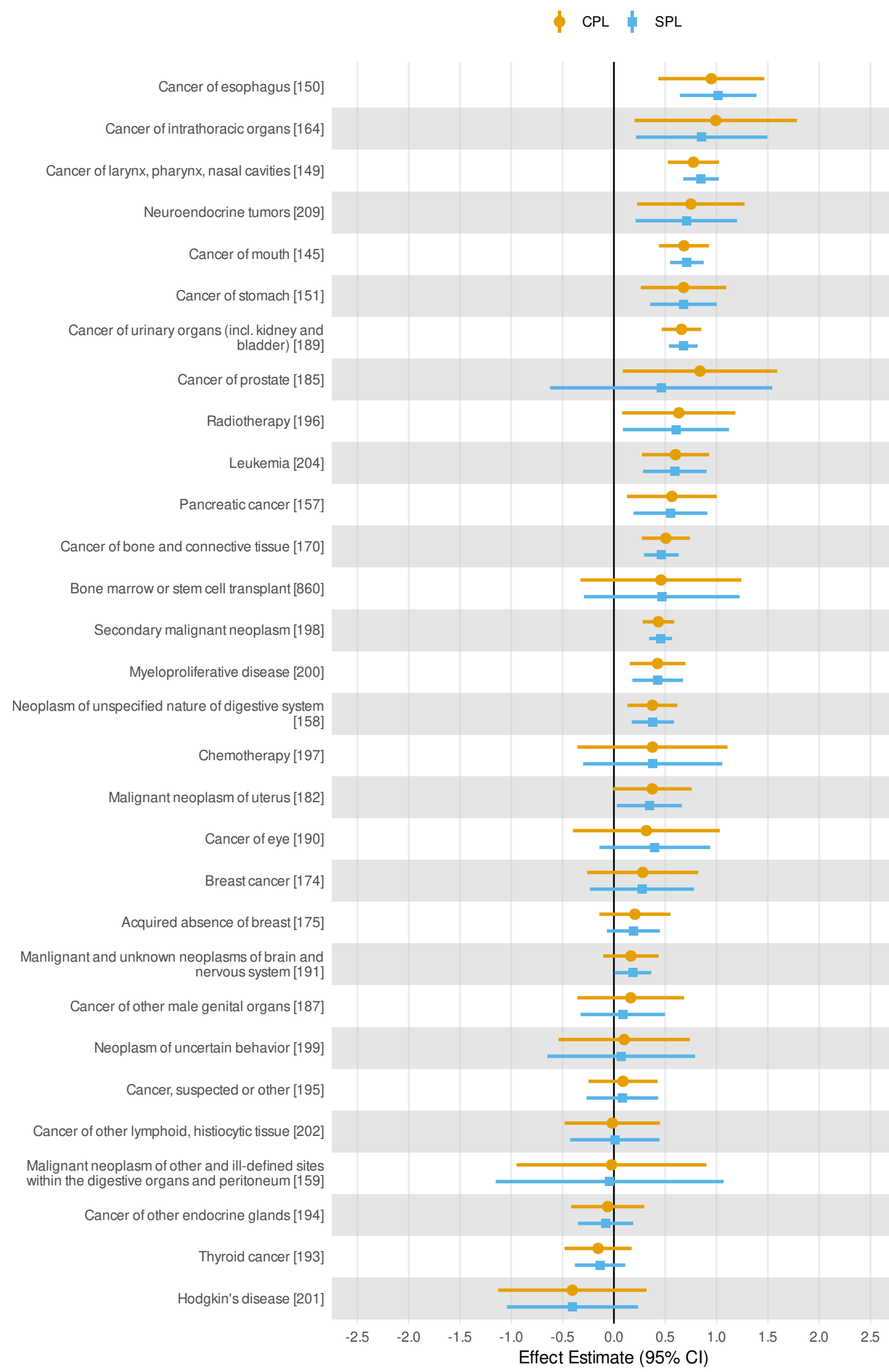

Figure S5: Association estimates between smoking and parent cancer types using two pseudo-likelihood methods: CPL (Centralized Pseudo-Likelihood) and SPL (Sequential Pseudo-Likelihood). Point estimates and 95% confidence intervals are shown for each method. The y-axis denotes cancer types and the x-axis represents the effect estimates. This analysis is performed using the multi-site MGI cohort, comprising the Anesthesiology, MIPACT, and MEND clinics.

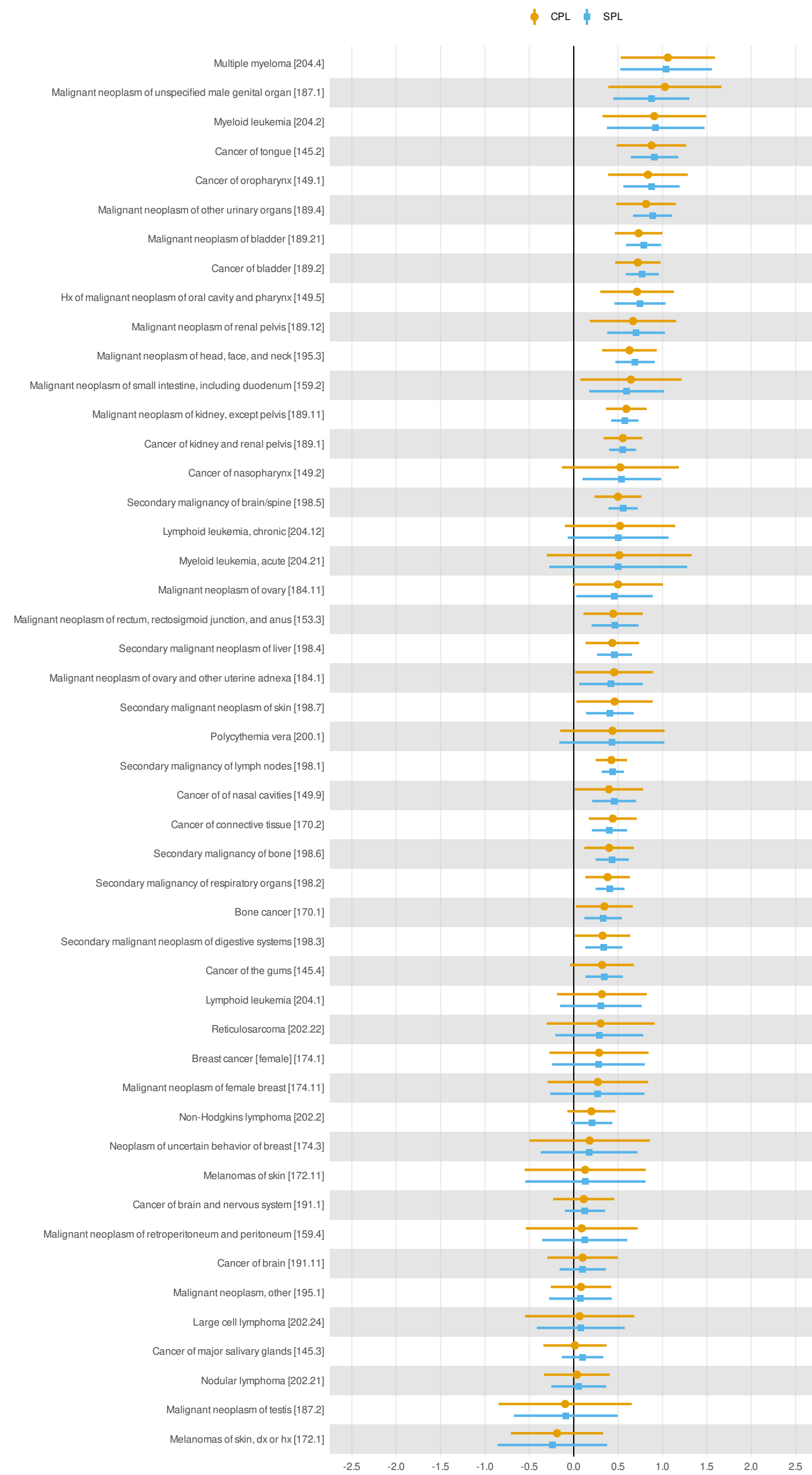

Figure S6: Association estimates between smoking and children cancer types using two pseudo-likelihood methods: CPL (Centralized Pseudo-Likelihood) and SPL (Sequential Pseudo-Likelihood). Point estimates and 95% confidence intervals are shown for each method. The y-axis denotes cancer types and the x-axis represents the effect estimates. This analysis is performed using the multi-site MGI cohort, comprising the Anesthesiology, MIPACT, and MEND clinics.
